# Draft genome of the Peruvian scallop *Argopecten purpuratus*

**DOI:** 10.1093/gigascience/giy031

**Published:** 2018-04-02

**Authors:** Chao Li, Xiao Liu, Bo Liu, Bin Ma, Fengqiao Liu, Guilong Liu, Qiong Shi, Chunde Wang

**Affiliations:** 1 Marine Science and Engineering College, Qingdao Agricultural University, Qingdao 266109, China; 2 Key Laboratory of Experimental Marine Biology, Institute of Oceanology, Chinese Academy of Sciences, Qingdao 266071, China; 3 Qingdao Oceanwide BioTech Co., Ltd., Qingdao 266101, China; 4 Shenzhen Key Lab of Marine Genomics, Guangdong Provincial Key Lab of Molecular Breeding in Marine Economic Animals, BGI Academy of Marine Sciences, BGI Marine, BGI, Shenzhen 518083, China

**Keywords:** *Argopecten purpuratus*, Peruvian scallop, genome assembly, annotation, gene prediction, phylogenetic analysis

## Abstract

**Background:**

The Peruvian scallop, *Argopecten purpuratus*, is mainly cultured in southern Chile and Peru was introduced into China in the last century. Unlike other *Argopecten* scallops, the Peruvian scallop normally has a long life span of up to 7 to 10 years. Therefore, researchers have been using it to develop hybrid vigor. Here, we performed whole genome sequencing, assembly, and gene annotation of the Peruvian scallop, with an important aim to develop genomic resources for genetic breeding in scallops.

**Findings:**

A total of 463.19-Gb raw DNA reads were sequenced. A draft genome assembly of 724.78 Mb was generated (accounting for 81.87% of the estimated genome size of 885.29 Mb), with a contig N50 size of 80.11 kb and a scaffold N50 size of 1.02 Mb. Repeat sequences were calculated to reach 33.74% of the whole genome, and 26,256 protein-coding genes and 3,057 noncoding RNAs were predicted from the assembly.

**Conclusions:**

We generated a high-quality draft genome assembly of the Peruvian scallop, which will provide a solid resource for further genetic breeding and for the analysis of the evolutionary history of this economically important scallop.

## Data Description

### Introduction

The Peruvian scallop (*Argopecten purpuratus*), also known as the Chilean scallop, is a medium-sized bivalve with a wide distribution in Peru and Chile [1]. In Chile, the cultured scallops reach a commercial size of around 9 cm in shell height within 14–16 months [2]. It is a relatively stenothermic species as its natural habitat is largely under the influence of upwelling currents from Antarctica [3]. Unlike other *Argopecten* scallops, the Peruvian scallop normally has a long life span of up to 7–10 years [4, 5]. This scallop was introduced into China in the late 2000s and has played an important role in stock improvement of *Argopecten* scallops via interspecific hybridization with bay scallops [6, 7].

### Whole genome sequencing

**Figure 1:**
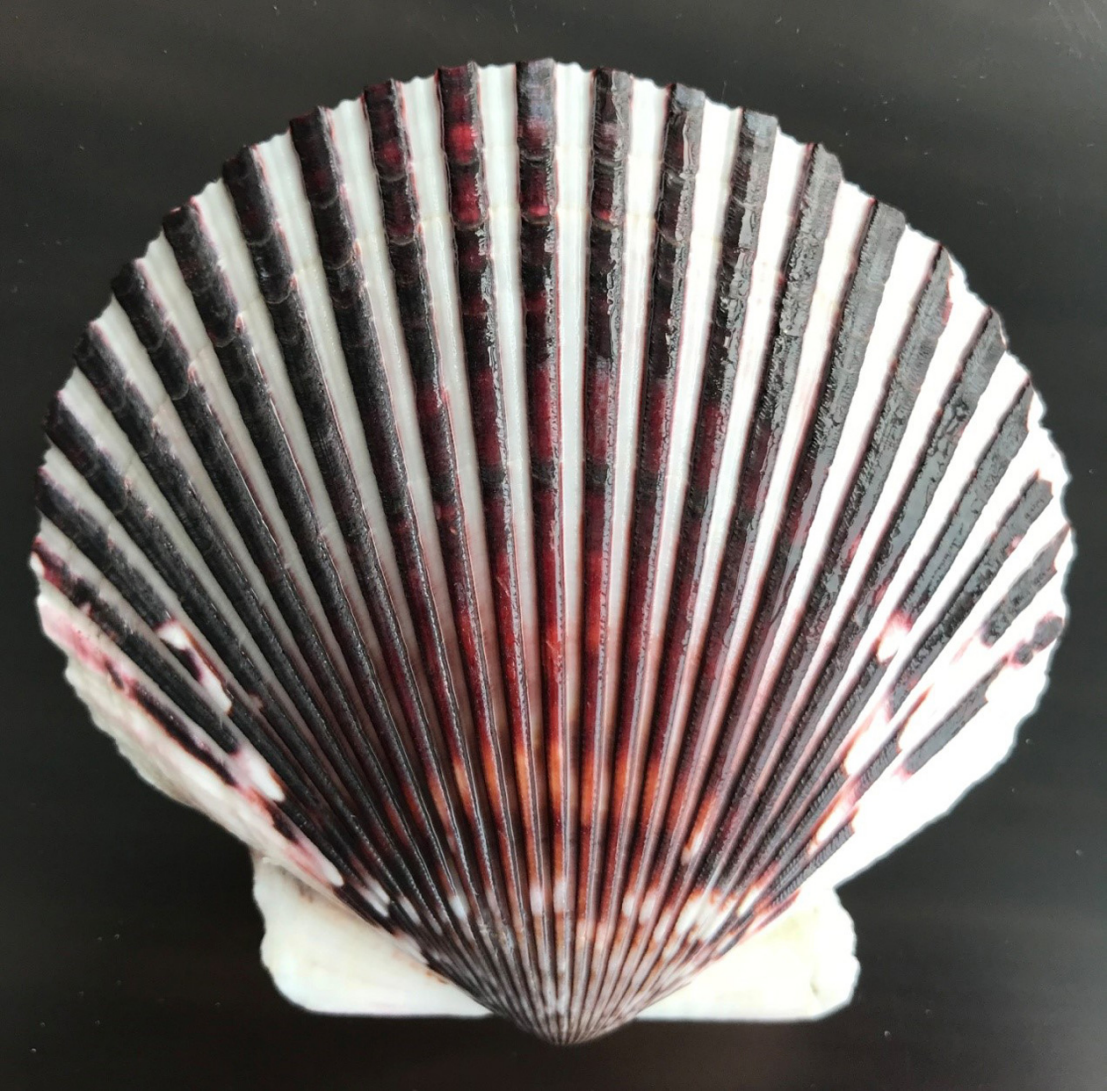
Picture of a representative Peruvian scallop in China.

Genomic DNA was extracted from an adductor muscle sample of a single *A. purpuratus* (Fig. 1), which was obtained from a local scallop farm in Laizhou, Shandong Province, China. A whole genome shotgun sequencing strategy was then applied. Briefly, six libraries with different insert length (250 bp, 450 bp, 2 kb, 5 kb, 10 kb, and 20 kb) were constructed according to the standard protocol provided by Illumina (San Diego, CA, USA). In detail, the DNA sample was randomly broken into fragments using covaris ultrasonic fragmentation apparatus. The library was prepared following end repair, adding sequence adaptor, purification, and polymerase chain reaction amplification. The mate-pair libraries (2 kb, 5 kb, 10 kb, and 20 kb) and paired-end libraries (250 bp, 450 bp) were all sequenced on the Illumina HiSeq4000 platform with paired-end 150 bp. In addition, SMRTbell libraries were prepared using either 10-kb or 20-kb preparation protocols. Briefly, the DNA sample was sheared by Diagenode Megaruptor2 (Belgium), the SMRTbell library was produced by ligating universal hairpin adapters onto double-stranded DNA fragments. Adapter dimers were efficiently removed using Pacific Biosciences' (PacBio's) MagBead kit. The final step of the protocol was to remove failed ligation products through the use of exonucleases. After the exonuclease and AMPure PB purification steps, sequencing primer was annealed to the SMRTbell templates, followed by binding of the sequence polymerase to the annealed templates. Subsequent sequencing was performed on PacBio Sequel instrument with Sequel^TM^ Sequencing Kit 1.2.1 (Pacific Biosciences, California, USA). Finally, the 10X Genomics library was constructed and sequenced with paired-end 150 bp on the Illumina Hiseq platform. The Chromium™ Genome Solution (10X Genomics, USA) massively partitions and molecularly bar codes DNA using microfluidics, producing sequencing-ready libraries with >1000,000 unique bar codes. In total, 463.19 Gb raw reads were generated, including 75.72, 70.22, 19.21, 45.71, 28.34, 11.78, 18.01, and 194.20 Gb from the 250-bp, 450-bp, 2-kb, 5-kb, 10-kb, and 20-kb libraries, PaBbio sequencing library, and 10X Genomics library, respectively. The raw reads were trimmed before being used for subsequent genome assembly. For Illumina HiSeq sequencing, the adaptor sequences, the reads containing more than 10% ambiguous nucleotides, as well as the reads containing more than 20% low-quality nucleotides (quality score less than 5),were all removed. For PacBio sequencing, the generated polymerase reads were first broken at the adaptor positions, and the subreads were generated after removal of the adaptor sequences. The subreads were then filtered by a minimum length = 50.

### Estimation of the genome size and sequencing coverage

The 17-mer frequency distribution analysis [8] was performed on the remaining clean reads to estimate the genome size of the Peruvian scallop using the following formula: genome size = k-mer number/peak depth. Based on a total number of 6.22 10^10^ k-mers and a peak k-mer depth of 69, the estimated genome size was calculated to be 885.29 Mb (Table 1) and the estimated repeat sequencing ratio was 33.74%.

**Table 1:** Summary of the Peruvian scallop genome assembly and annotation

Genome assembly	Parameter
Contig N50 size (kb)	80.11
Scaffold N50 size (Mb)	1.02
Estimated genome size (Mb)	885.29
Assembled genome size (Mb)	724.78
Genome coverage ()	303.83
Longest scaffold (bp)	11,125,,544
Genome annotation	Parameter
Protein-coding gene number	26,256
Average transcript length (kb)	10.53
Average CDS length (bp)	1,418.29
Average intron length (bp)	1,505.92
Average exon length (bp)	201.09
Average exons per gene	7.05

### *De novo* genome assembly and quality assessment of *A. purpuratus* genome

All the pair-end Illumina reads were first assembled into scaffolds using Platanus_v1.2.4 (Platanus, RRID:SCR_015531) [9], and the gaps were then filled by GapCloser_v1.12-r6 (GapCloser, RRID:SCR_015026) [10]. Subsequently, the PacBio data were used for additional gap filling by PBJelly_v14.1 (PBJelly, RRID:SCR_012091) with default parameters [11], and then all of the Illumina reads were used to correct the genome assembly by Pilon_v1.18 (Pilon, RRID:SCR_014731) for two rounds [12]. After that, the 10X linked-reads were used to link scaffolds by fragScaff_140 324.1 [13]. First, in order to solve the issue of heterozygosity, in our assembly process we chose 19-kmer to draw k-mer distribution histogram and classified all the kmers into homozygous kmer and heterozygous kmer according to the coverage depth. Second, we utilized 45-kmer to construct the de Bruijn figure and combine the bubbles for heterozygous sites, according to the sequences with longer length and deeper coverage depth. Then, the pair-end information was used to determine the connection between the heterozygous parts and filter the contigs lacking support. Finally, the heterozygous contigs and homozygous contigs were distinguished based on contig coverage depth. After assembly, the reads from short insert length libraries were mapped onto the assembled genome. Only one peak was observed in the sequencing depth distribution analysis with the average sequencing depth of 148.2×, which is consistent with the sequencing depth, indicating high quality of the assembled scallop genome. Finally, a draft genome of 724.78 Mb was assembled (accounting for 81.87% of the estimated genome size of 885.29 Mb), with a contig N50 size of 80.11 kb and scaffold N50 size of 1.02 Mb (Table1).

With this initial assembly, we mapped the short insert library reads onto the assembled genome using BWA_0.6.2 (BWA, RRID:SCR_010910) software [14] to calculate the mapping ratio and assess the assembly integrity. In summary, 91.05% of the short reads were mapped onto the assembled genome with a coverage of 89.40%, indicating high reliability of genome assembly. CEGMA_v2.5 (Core Eukaryotic Genes Mapping Approach; CEGMA, RRID:SCR_015055) defines a set of conserved protein families that occur in a wide range of eukaryotes and presents a mapping procedure to accurately identify their exon-intron structures in a novel genomic sequence [15]. A protein is classified as complete if the alignment of the predicted protein to the HMM profile represents at least 70% of the original KOG domain, or otherwise classified as partial. Through mapping to the 248 core eukaryotic genes, 222 genes (89.52%) were identified. BUSCO_v3 (Benchmarking Universal Single-Copy Orthologs; RRID:SCR_015008) provides quantitative measures for the assessment of genome assembly completeness, based on evolutionarily informed expectations of gene content from near-universal single-copy orthologs [16]. We confirmed that 89% of the 843 single-copy genes were identified, indicating good integrity of the genome assembly.

### Repeat sequence analysis of the genome assembly

We searched transposable elements in the assembled genome through *ab-initio* and homology-based methods. For the first method, we applied RepeatModeler_1.0.4 (RepeatModeler, RRID:SCR_015027) [17] (the parameter set as “–engine_db wublast”) to build a specific repeat database. For the second method, we used known repeat library (Repbase) [18] to identify repeats with RepeatMasker_open-4.0 [19] (the parameter set as “-a -nolow -no_is -norna -parallel 3 -e wublast –pvalue 0.0001”) and RepeatProteinMask (the parameter set as “-noLowSimple -pvalue 0.0001 -engine wublast”) [19]. Tandem repeats finder_4.04 (TRF) was used to find tandem repeats with the parameters setting as “Match = 2, Mismatching penalty = 7, Delta = 7, PM = 80, PI = 10, Minscore = 50, MaxPeriod = 2000” [20]. Finally, we determined that the total repeat sequences are 294,496,811 bp, accounting for 40.63% of the assembled genome, and including 11.46% of tandem repeats, which is consistent with our above-mentioned estimation (Table2).

**Table 2:** The prediction of repeat elements in the Peruvian scallop genome

Type	Repeat size (bp)	% of genome
TRF	83,037,380	11.46
RepeatMasker	237,471,691	32.76
RepeatProteinMask	21,719,425	3.00
Total	294,496,811	40.63

### Gene annotation

#### Annotation of protein coding genes

The annotation strategy for protein-coding genes integrated *de novo* prediction with homology and transcriptome data-based evidence. Homology sequences from African malaria mosquito (*Anopheles gambiae*), ascidian (*Ciona intestinalis*), Florida lancelet (*Branchiostoma floridae*), fruit fly (*Drosophila melanogaster*), human (*Homo sapiens*), leech (*Helobdella robusta*), nematode (*Caenorhabditis elegans*), octopus (*Octopus bimaculoides*), owl limpet (*Lottia gigantea*), Pacific oyster (*Crassostrea gigas*), and sea urchin (*Strongylocentrotus purpuratus*) were downloaded from Ensemble [21]. The protein sequences of homology species were aligned to the assembled genome with TBLASTn (Basic Local Alignment Search Tool; e-value ≤10^−5^) [22], and gene structures were predicted with GeneWise_2.4.1 (GeneWise, RRID:SCR_015054) (the parameter set as “-genesf”) [23]. The transcriptome data were generated from adductor muscle, hepatopancreas, and mantle on Illumina HiSeq4000 platform. Tophat_2.1.1 (the parameter set as “–max-intron-length 500 000 -m 2 –library-type fr-unstranded”) [24] was utilized to map the transcriptome data onto genome assembly and then Cufflinks_2.1.0 (Cufflinks, RRID:SCR_014597), the parameter set as “–multi-read-correct”[25], was used to generate gene model according to the pair-end relationships and the overlap between aligned reads. The *de novo* prediction of genes was carried out with four programs: Augustus_3.0.3 (Augustus: Gene Prediction, RRID:SCR_008417), the parameter set as “-uniqueGeneId true –noInFrameStop = true –gff3 on –genemodel complete –strand both” [26]; GENSCAN (GENSCAN, RRID:SCR_012902), with default parameter [27]; GlimmerHMM_3.0.2 (GlimmerHMM, RRID:SCR_002654), the parameter set as " -f -g" [28]; and SNAP (the default parameter) [29]. All evidences of the gene model were integrated using EvidenceModeler_r2012-06-25 (EVM) [29]. Finally, we identified 26,256 protein-coding genes in the Peruvian scallop genome. In detail, 26,513 genes were predicted through the *de novo* method, 19,394 genes were annotated by RNA transcripts or raw RNA reads, and 15,608 genes were supported by homolog evidences. The average transcript length, CDS length, and intron length were 10,534 bp, 1,418 bp, and 1,505 bp, respectively (Table1).

#### Gene functional annotation

Gene functions were predicted from the best BLASTP (e-value ≤10^−5^) hits in SwissProt databases [30]. Gene domain annotation was performed by searching the InterPro (InterPro, RRID:SCR_006695) database [31]. All genes were aligned against Kyoto Encyclopedia of Genes and Genomes (KEGG, RRID:SCR_012773) [32] to identify the best hits for pathways. Gene ontology terms for genes were obtained from the corresponding InterPro entry [33]. Finally, among these annotated genes, 70.3% of encoded proteins showed homology to proteins in the SwissProt database, 91.1% were identified in the nonredundant database, 70.4% were identified in the KEGG database, 72.1% were identified in the InterPro database, and 92.1% could be mapped onto the functional databases.

#### Noncoding RNA annotation

The noncoding RNA genes, including miRNAs, rRNAs, snRNAs, and tRNAs, were identified. The tRNAscan-SE_2.0 (tRNAscan-SE, RRID:SCR_010835) software with eukaryote parameters [34] was used to predict tRNA genes. The miRNA and snRNA genes in the assembled genome were extracted by INFERNAL_1.1.2 software [35] against the Rfam (Rfam, RRID:SCR_007891) database [36] with default parameters. Finally, 1132 miRNAs, 1664 tRNAs, 41 rRNAs, and 220 snRNAs were discovered from the Peruvian scallop genome.

### Global gene family classification

Protein-coding genes from the Peruvian scallop and other sequenced species, including Brachiopod (*Lingula anatina*), brown mussel (*Modiolus philippinarum*), California sea hare (*Aplysia californica*), cold seep mussel (*Bathymodiolus platifrons*), Florida lancelet (*B. floridae*), fruit fly (*D. melanogaster*), human (*H. sapiens*), leech (*H. robusta, Capitella teleta*), nematode (*C. elegans*), octopus (*O. bimaculoides*), owl limpet (*L. gigantea*), Pacific abalone (*Haliotis discus*), Pacific oyster (*C. gigas*), pearl oyster (*Pinctada fucata*), red flour beetle (*Tribolium castaneum*), and Yesso scallop (*Patinopecten yessoensis*) were analyzed. All data were downloaded from Ensemble [21] or National Center for Biotechnology Information (NCBI) [37]. For each protein-coding gene with alternative splicing isoforms, only the longest protein sequence was kept as the representative.

**Figure 2:**
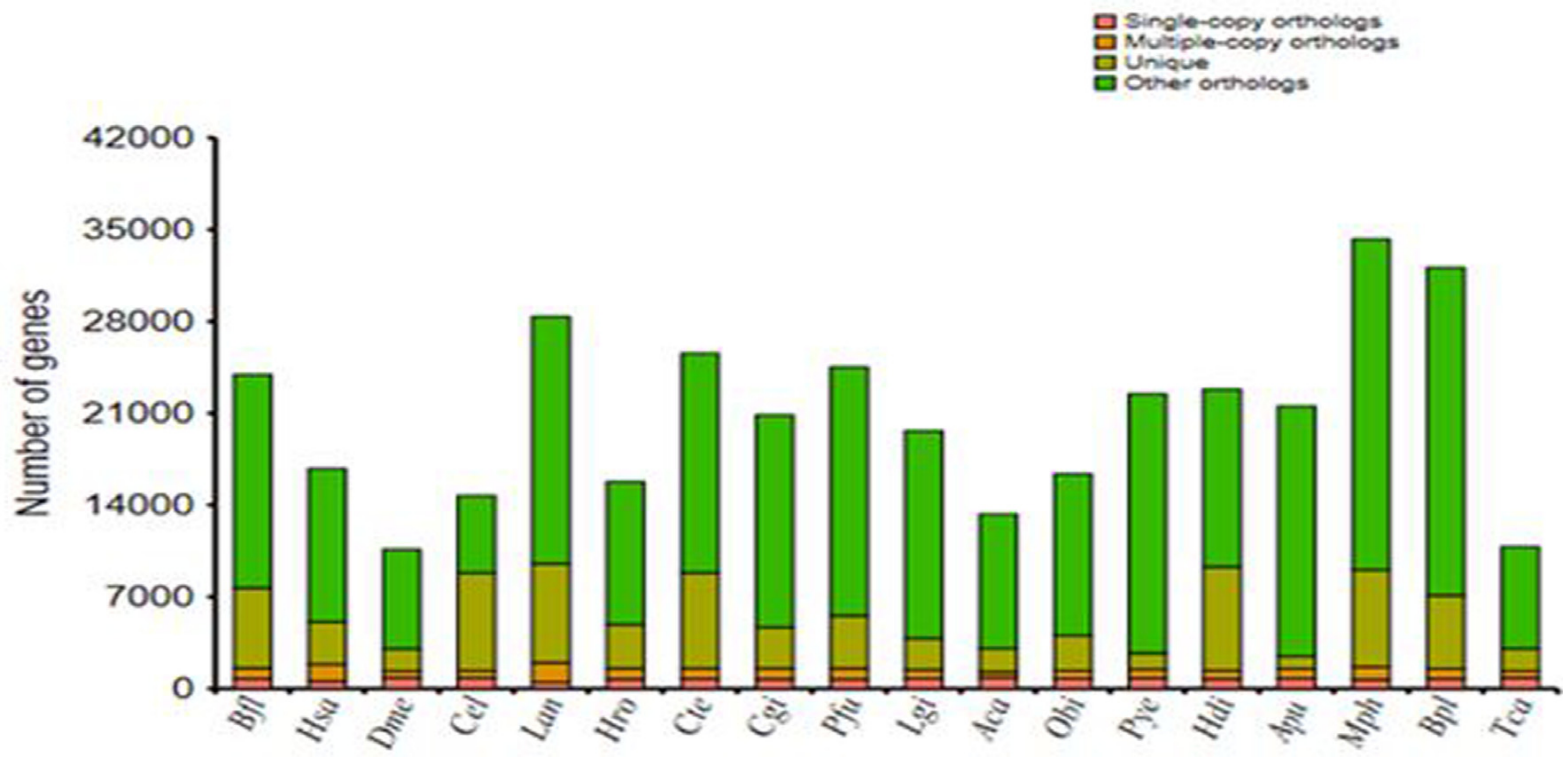
Distribution of genes in different species. Abbreviations: Aca, *Aplysia californica;* Apu, *Argopecten purpuratus;* Bfl, *Branchiostoma floridae;* Bpl, *Bathymodiolus platifrons;* Cel, *Caenorhabditis elegans;* Cgi, *Crassostrea gigas; Cte, Capitella teleta;* Dme, *Drosophila melanogaster;* Hsa, *Homo sapiens;* Hdi, *Haliotis discus;* Hro, *Helobdella robusta;* Lan, *Lingula anatina;* Lgi, *Lottia gigantea;* Mph, *Modiolus philippinarum;* Obi, *Octopus bimaculoides;* Pfu, *Pinctada fucata;* Pye, *Patinopecten yessoensis*; Tca, *Tribolium castaneum*.

Gene family analysis based on the homolog of gene sequences in related species was initially implemented by the alignment of an “all against all” BLASTP (with a cutoff of 1e-7) and subsequently followed by alignments with high-scoring segment pairs conjoined for each gene pair by TreeFam_3.0 [38]. To identify homologous gene pairs, we required more than 30% coverage of the aligned regions in both homologous genes. Finally, homologous genes were clustered into gene families by OrthoMCL-5 (OrthoMCL DB: Ortholog Groups of Protein Sequences, RRID:SCR_007839) [39] with the optimized parameter of “-inflation 1.5.”All protein-coding genes from the 18 examined genomes were used to assign gene families. In total, the protein-coding genes were classified into 45,268 families and 108 strict single-copy orthologs (Fig.2).

### Phylogenetic analysis

**Figure 3:**
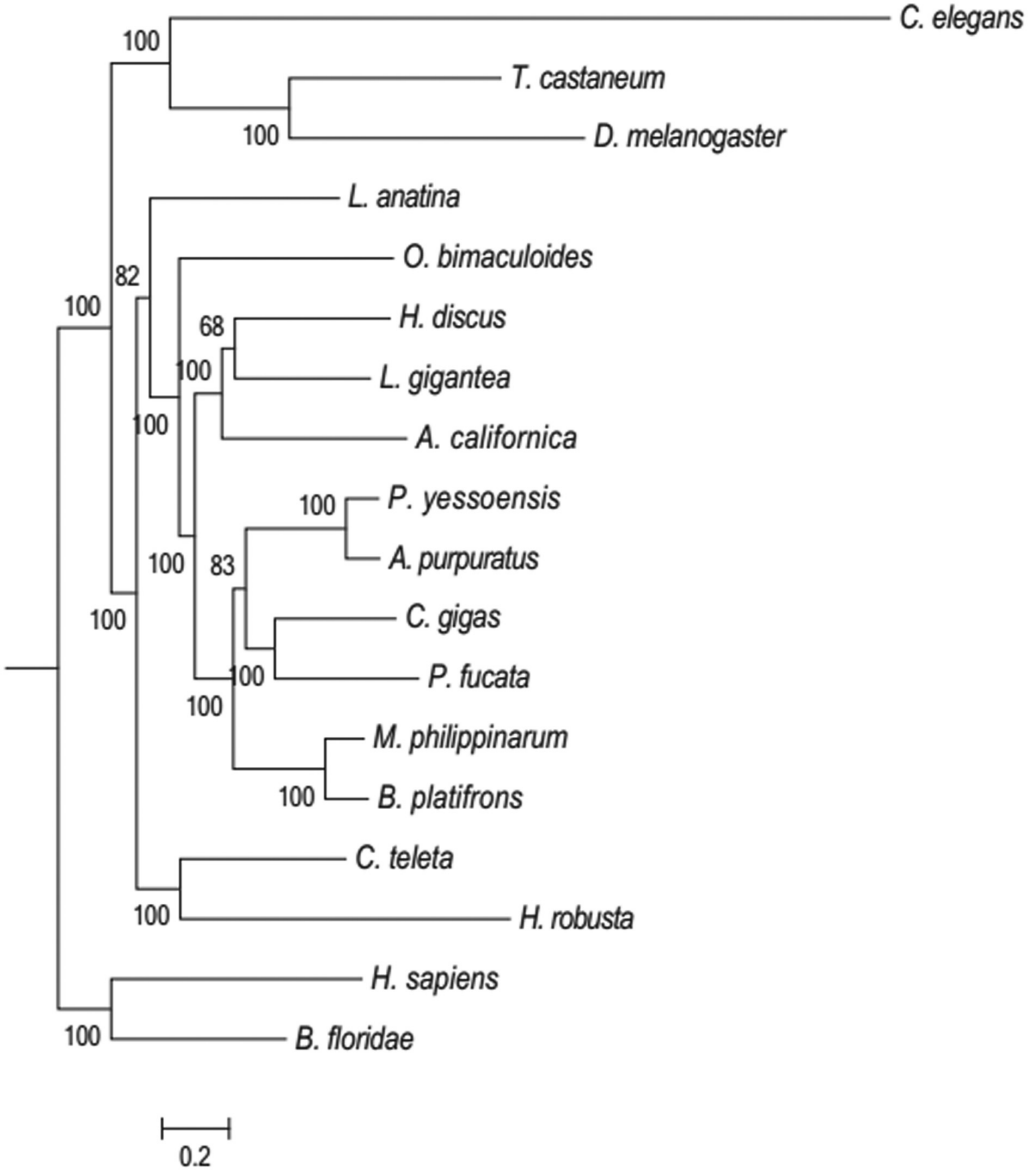
Bootstrap support of phylogenetic tree. A maximum likelihood tree was constructed using RAxML based on 108 single-copy protein-coding genes of the related species. The total number of bootstrap was 100.

Evolutionary analysis was performed using these single-copy protein-coding genes from the 18 examined species. Amino acid and nucleotide sequences of the ortholog genes were aligned using the multiple alignment software MUSCLE (MUSCLE, RRID:SCR_011812) with default parameters [40]. A total number of 108 single-copy ortholog alignments were concatenated into a super alignment matrix of 242,085 nucleotides. A maximum likelihood method deduced tree was inferred based on the matrix of nucleotide sequences using RAxML_v8.0.19 (RAxML, RRID:SCR_006086) with default nucleotide substitution model-PROTGAMMAAUTO [41]. Clade support was assessed using bootstrapping algorithm in the RAxML package with 100 alignment replicates (Fig. 3) [42]. The constructed phylogenetic tree (Fig. 3) indicated that the Peruvian scallop and Yesso scallop were clustered closely first and then clustered with oysters and mussels, which is in consistent with their putative evolution relationships [43-46].

### The estimation of divergence time

The species divergence times were inferred with MCMCTree included in PAML v4.7a (PAML, RRID:SCR_014932) [47] with the parameter set as “burn-in = 1000, sample-number = 1000 000, sample-frequency = 2,”and evolutionary analysis was performed using single-copy protein-coding genes from the 18 examined species. Based on the phylogenetic tree (Fig.3), the molecular clock was calibrated based on the fossil records according to previous studies [48-50]. Finally, we estimated that the divergence between the Peruvian scallop and Yesso scallop happened at 113.6 million years ago.

## Conclusions

In the present study, we report the first whole genome sequencing, assembly, and annotation of the Peruvian scallop (*A. purpuratus*), an economically important bivalve in Chile, Peru, and China. The assembled draft genome of 724.78 Mb accounts for 81.87% of the estimated genome size (885.29 Mb). A total of 26,256 protein-coding genes and 3,057 noncodingRNAs were predicted from the genome assembly. This genome assembly will provide solid support for in-depth biological studies. With the availability of these genomic data, subsequent development of genetic markers for further genetic selection and molecular breeding of scallops could be realized. The current genome data will also facilitate genetic analyses of the evolutionary history of the abundant scallops in the world.

## Availability of supporting data

Supporting data are available in the *GigaScience* database [52]. Raw data have been deposited in NCBI with the project accession PRJNA418203. BioSample accessions: SAMN08022140 (genome); SAMN08731415 (transcriptome; muscle); SAMN08731411 (transcriptome; mantle); and SAMN08731410 (transcriptome; hepatopancreas).

## Abbreviations

BLAST: Basic Local Alignment Search Tool; KEGG: Kyoto Encyclopedia of Genes and Genomes; NCBI: National Center for Biotechnology Information.

## Competing interests

The authors declare that they have no competing interests.

## Funding

This work was supported by the National Natural Science Foundation of China (31572618 grant to C.W. and 41676152 grant to X.L.) and the earmarked fund for Shandong Modern Agro-Industry Technology Research System (SDAIT-14) grant to C.W.

## Author contributions

C.W., X.L., and C.L. designed the project. B.M., F.L., and G.L. collected the samples and prepared the quality control. C.L., C.W., and X.L. were involved in the data analyses. C.W., X.L., C.L., and Q.S. wrote the manuscript. All authors read and approved the final manuscript.

## Supplementary Material

GIGA-D-17-00315_Original_Submission.pdf

GIGA-D-17-00315_Revision_1.pdf

GIGA-D-17-00315_Revision_2.pdf

Response_to_Reviewer_Comments_Original_Submission.pdf

Response_to_Reviewer_Comments_Revision_1.pdf

Reviewer_1_Report_(Original_Submission) -- Kevin Kocot 12/29/2017 Reviewed

Reviewer_2_Report_(Original_Submission) -- Takeshi Takeuchi, Ph.D 1/4/2018 Reviewed

Reviewer_2_Report_(Revision_1) -- Takeshi Takeuchi, Ph.D 2/14/2018 Reviewed
