# Supplementary material for: Draft genome of the Peruvian scallop Argopecten purpuratus
Source: Gigascience. 2018 Apr 2;7(4):giy031. doi: 10.1093/gigascience/giy031 (PMC5905365; doi:10.1093/gigascience/giy031)
Supplement: GIGA-D-17-00315_Revision_1.pdf [file giy031_giga-d-17-00315_revision_1.pdf]

# GigaScience


## Draft genome of the Peruvian scallop Argopecten purpuratus

--Manuscript Draft--

| | |
|---|---|
| Manuscript Number: | GIGA-D-17-00315R1 |
| Full Title: | Draft genome of the Peruvian scallop Argopecten purpuratus |
| Article Type: | Data Note |
| Funding Information: | National Natural Science Foundation of China (31572618) — Dr. Chunde Wang<br>National Natural Science Foundation of China (41676152) — Dr. Xiao Liu<br>Fund for Shandong Modern Agro-Industry Technology Research System (SDAIT-14) — Dr. Chunde Wang |
| Abstract: | Background: The Peruvian scallop, Argopecten purpuratus, is mainly cultured in Southern Chile and had been introduced into China in last century. Unlike other Argopecten scallops, the Peruvian scallop normally has a long life span of up to 7-10 years. Therefore, researchers have been employing it to develop hybrid vigor. Here, we performed whole genome sequencing, assembly, and gene annotation of the Peruvian scallop, with an important aim to develop genomic resources for genetic breeding in scallops. Findings: A total of 463.19-Gb (Gigabase) raw DNA reads were sequenced. The draft genome assembly of 724.78 Mb was generated (accounting for 81.87% of the estimated genome size 885.29 Mb), with a contig N50 size of 80.11 kb and scaffold N50 size of 1.02 Mb. Meanwhile, the repeat sequences were calculated to reach 33.74% of the whole genome, and a total of 26,256 protein-coding genes and 3,057 non-coding RNAs were predicted from the assembly. Conclusion: We generated a draft genome assembly of the Peruvian scallop, which will provide solid resource for further genetic breeding and evolutionary history analysis of this economically important scallop. |
| Corresponding Author: | Chunde Wang, Ph.D<br>Qingdao Agricultural University<br>CHINA |
| Corresponding Author Secondary Information: | |
| Corresponding Author's Institution: | Qingdao Agricultural University |
| Corresponding Author's Secondary Institution: | |
| First Author: | Chao Li, Ph.D |
| First Author Secondary Information: | |
| Order of Authors: | Chao Li, Ph.D<br>Xiao Liu, D.Sc<br>Bo Liu, D.Sc<br>Bin Ma, MSc<br>Guilong Liu, MSc<br>Qiong Shi, Ph.D<br>Chunde Wang, Ph.D |
| Order of Authors Secondary Information: | |
| Response to Reviewers: | Dear Editor,<br>Thanks for your kindly consideration of publication of our manuscript in the journal. We |

Powered by Editorial Manager® and ProduXion Manager® from Aries Systems Corporation

| | |
|---|---|
| | appreciate the insightful comments from the anonymous reviewers very much. I have inlcuded the response letter in the supplementary file as the figures will not show up in this box. If you have more questions, please feel free to let us know in your earliest convenience.<br><br>Best regards,<br><br>Chao and Chunde |
| **Additional Information:** | |
| **Question** | **Response** |
| Are you submitting this manuscript to a special series or article collection? | No |
| **Experimental design and statistics**<br><br>Full details of the experimental design and statistical methods used should be given in the Methods section, as detailed in our Minimum Standards Reporting Checklist. Information essential to interpreting the data presented should be made available in the figure legends.<br><br>Have you included all the information requested in your manuscript? | Yes |
| **Resources**<br><br>A description of all resources used, including antibodies, cell lines, animals and software tools, with enough information to allow them to be uniquely identified, should be included in the Methods section. Authors are strongly encouraged to cite Research Resource Identifiers (RRIDs) for antibodies, model organisms and tools, where possible.<br><br>Have you included the information requested as detailed in our Minimum Standards Reporting Checklist? | Yes |
| **Availability of data and materials**<br><br>All datasets and code on which the conclusions of the paper rely must be either included in your submission or deposited in publicly available repositories (where available and ethically appropriate), referencing such data using a unique identifier in the references and in the "Availability of Data and Materials" section of your manuscript. | Yes |

Powered by Editorial Manager® and ProduXion Manager® from Aries Systems Corporation

| | |
|---|---|
| Have you have met the above requirement as detailed in our Minimum Standards Reporting Checklist? | |

Powered by Editorial Manager® and ProduXion Manager® from Aries Systems Corporation

Manuscript

Click here to download Manuscript ZS-manuscript-final20180124.docx

# Draft genome of the Peruvian scallop *Argopecten purpuratus*

Chao Li[1], Xiao Liu[2], Bo Liu[1], Bin Ma[3], Guilong Liu[1], Qiong Shi[4], Chunde Wang[1,*]

[1]Marine Science and Engineering College, Qingdao Agricultural University, Qingdao 266109, China

[2]Key Laboratory of Experimental Marine Biology, Institute of Oceanology, Chinese Academy of Sciences, Qingdao 266071, China

[3]Qingdao Oceanwide BioTech Co., Ltd., Qingdao 266101, China

[4]Shenzhen Key Lab of Marine Genomics, Guangdong Provincial Key Lab of Molecular Breeding in Marine Economic Animals, BGI Academy of Marine Sciences, BGI Marine, BGI, Shenzhen 518083, China

[*]Correspondence address. Professor Chunde Wang, Marine Science and Engineering College, Qingdao Agricultural University, Qingdao 266109, China (tel: +8613589227997; email: chundewang2007@163.com)

Email addresses of all authors:

Chao Li, leoochao@163.com

Xiao Liu, liuxiao@qdio.ac.cn

Bo Liu, liubomusic@126.com

Bin Ma, hereiammabin@163.com

Guilong Liu, 969613442@qq.com

Qiong Shi, shiqiong@genomics.cn

**Abstract**

**Background:** The Peruvian scallop, *Argopecten purpuratus*, is mainly cultured in Southern Chile and had been introduced into China in last century. Unlike other *Argopecten* scallops, the Peruvian scallop normally has a long life span of up to 7-10 years. Therefore, researchers have been employing it to develop hybrid vigor. Here, we performed whole genome sequencing, assembly, and gene annotation of the Peruvian scallop, with an important aim to develop genomic resources for genetic breeding in scallops. **Findings:** A total of 463.19-Gb (Gigabase) raw DNA reads were sequenced. The draft genome assembly of 724.78 Mb was generated (accounting for 81.87% of the estimated genome size 885.29 Mb), with a contig N50 size of 80.11 kb and scaffold N50 size of 1.02 Mb. Meanwhile, the repeat sequences were calculated to reach 33.74% of the whole genome, and a total of 26,256 protein-coding genes and 3,057 non-coding RNAs were predicted from the assembly. **Conclusion:** We generated a draft genome assembly of the Peruvian scallop, which will provide solid resource for further genetic breeding and evolutionary history analysis of this economically important scallop.

**Keywords:** *Argopecten purpuratus*; Peruvian scallop; genome assembly; annotation; gene prediction; phylogenetic analysis

## Data description

### Introduction

The Peruvian scallop (*Argopecten purpuratus*), also known as the Chilean scallop, is a medium-sized bivalve with a wide distribution in Peru and Chile [1]. In Chile, the cultured scallops reach a commercial size of around 9 cm in shell height within 14-16 months [2]. It is a relatively stenothermic species as its natural habitat is largely under the influence of upwelling currents from Antarctica [3]. Unlike other *Argopecten* scallops, the Peruvian scallop normally has a long life span of up to 7-10 years [4, 5]. This scallop was introduced into China in the late 2000's and had played an important role in stock improvement of *Argopecten* scallops via inter-specific hybridization [6, 7] with bay scallops.

### Whole genome sequencing

Genomic DNA was extracted from adductor muscle sample of a single *A. purpuratus* (Figure 1), which was obtained from a local scallop farm in Laizhou, Shandong Province, China. A whole genome shotgun sequencing strategy was applied. Six libraries with different insert length (250 bp, 450 bp, 2 kb, 5 kb, 10 kb, and 20 kb) were constructed according to the standard protocol provided by Illumina (San Diego, CA, USA). Briefly, the DNA sample was randomly broken into fragments by Covaris ultrasonic fragmentation apparatus. The library was prepared following end repair, adding sequence adaptor, purification, and PCR amplification. The mate-pair libraries (2 kb, 5 kb, 10 kb, and 20 kb) and paired-end libraries (250 bp, 450 bp) were all sequenced on Illumina HiSeq4000 platform with paired-end 150 bp. In addition,

SMRTbell libraries were prepared using either 10-kb or 20-kb preparation protocols. Briefly, the DNA sample was sheared by Diagenode Megaruptor2 (the Kingdom Of Belgium), the SMRTbell library was produced by ligating universal hairpin adapters onto double-stranded DNA fragments. Adapter dimers were efficiently removed using PacBio's MagBead kit. The final step of the protocol was to remove failed ligation products through the use of exonucleases. After the exonuclease and AMPure PB purification steps, sequencing primer was annealed to the SMRTbell templates, followed by binding of the sequence polymerase to the annealed templates. Subsequent sequencing was performed on PacBio Sequel instrument with Sequel™ Sequencing Kit 1.2.1 ( Pacific Biosciences of California，USA ). Finally, the 10X Genomics library was constructed and sequenced with paired-end 150 bp on the Hiseq platform. The Chromium™ Genome Solution（10X Genomics，USA）massively partitions and molecularly barcodes DNA using microfluidics, producing sequencing-ready libraries with >1,000,000 unique barcodes. In total, 463.19 gigabases (Gb) of raw reads were generated, including 75.72, 70.22, 19.21, 45.71, 28.34, 11.78, 18.01 and 194.20 Gb from the 250-bp, 450-bp, 2-kb, 5-kb, 10-kb, 20-kb libraries, Pacbio sequencing, and 10X Genomics library respectively. The raw reads were trimmed before used for subsequent genome assembling. For Illumina HiSeq sequencing, the adaptor sequences, the reads contain more than 10% ambiguous nucleotides, as well as the reads contains more than 20% of the low quality nucleotides (quality score less than 5) were all removed. For PacBio sequencing, the generated polymerase reads were firstly broke at the adaptor position, the subreads were generated following removing the adaptor sequences. Then the subreads were filtered by minimum length = 50.

Estimation of the genome size and sequencing coverage

The 17-mer frequency distribution analysis [8] was performed on all the remaining clean reads to estimate the genome size of the Peruvian scallop using the following formula: genome size = k-mer number / peak depth. A total number of $6.22 \times 10^{10}$ k-mers and the peak k-mer depth of 69 was employed to obtain the estimated genome size at 885.29 Mb (Table 1), and the estimated repeat sequencing ratio reaches 33.74%.

*De novo* genome assembly and quality assessment of *A. purpuratus* genome

All the pair-end Illumina reads were first assembled into scaffolds using Platanus_v1.2.4 [9], and then were applied to fill the gaps by GapCloser_v1.12-r6 [10]. Subsequently, the Pacbio data were used for additional gap filling by PBJelly_v14.1 with default parameters [11], and then all the Illumina reads were employed for two rounds to correct the genome assembly by Pilon_v1.18 [12]. After that, the 10X linked-reads were used to link scaffolds by fragScaff_140324.1 [13]. Particularly, in order to solve the issue of heterozygosity, in our assembly process, we chose 19-kmer to draw k-mer distribution histogram, and classified all the kmers into homozygous kmer and heterozygous kmer according to the coverage depth at first. Secondly, we utilized 45-kmer to construct the de bruijn figure, combine the bubbles for heterozygous sites, according to the sequences with longer length and deeper coverage depth. Then, the reads information and pair-end information were used to determine the connection between the heterozygous parts, and construct the contigs, filtered the contigs lacking support. Finally, the heterozygous contigs and homozygous contigs were distinguished through contigs coverage depth information. After assembly, the reads from short insert length libraries were mapped to the assembled genome. And only one peak was observed in the sequencing depth

distribution analysis with the average sequencing depth 148.2 X, which is consistent with the sequencing depth, indicated high quality of the assembled scallop genome. Finally, a draft genome of 724.78 Mb was assembled (accounting for 81.87% of the estimated genome size at 885.29 Mb), with a contig N50 size of 80.11 kb and a scaffold N50 size of 1.02 Mb (Table 1).

With this initial assembly, we applied the short insert library reads to map with the assembled genome using BWA_0.6.2 software [14] to calculate the mapping ratio and assess the assembly integrity. In summary, 91.05% short reads were mapped onto the assembled genome with a coverage of 89.40%, indicating a good reliability of our genome assembly. CEGMA_v2.5 (Core Eukaryotic Genes Mapping Approach) defines a set of conserved protein families that occur in a wide range of eukaryotes, and presents a mapping procedure to accurately identify their exon-intron structures in a novel genomic sequence [15]. A protein is classified as complete if the alignment of the predicted protein to the HMM profile represents at least 70% of the original KOG domain, otherwise it is classified as partial. Through mapping to the 248 core eukaryotic genes, a total of 222 genes (89.52%) were identified. BUSCO_v3 (Benchmarking Universal Single-Copy Orthologs) provides quantitative measures for the assessment of genome assembly completeness, based on evolutionarily-informed expectations of gene content from near-universal single-copy orthologs [16]. We confirmed that 89% of the 843 single-copy genes were identified, indicating a good integrity of the genome assembly.

Repeat sequence analysis of the genome assembly

We searched transposable elements (TEs) in the assembled genome through *ab-initio* and homology based methods. For the former method, we applied

RepeatModeler_1.0.4 [17] (the parameter set as '--engine_db wublast') to build a specific repeat database. For the latter method, we employed known repeat library (Repbase) [18] to identify repeats with RepeatMasker_open-4.0 [19] (the parameter set as '-a -nolow -no_is -norna -parallel 3 -e wublast --pvalue 0.0001') and RepeatProteinMask (the parameter set as '-noLowSimple -pvalue 0.0001 -engine wublast') [19]. Tandem repeats finder_4.04 (TRF) was used to find tandem repeats with the parameters setting as 'Match = 2, Mismatching penalty = 7, Delta = 7, PM = 80, PI = 10, Minscore = 50, MaxPeriod = 2,000' [20]. Finally, we summarized that the total repeat sequences are 294,496,811 bp, accounting for 40.63% of the assembled genome, and including 11.46% of tandem repeats, which is consistent with our above-mentioned estimation (Table 2).

Gene annotation

*(1) Annotation of protein coding genes*

The annotation strategy for protein-coding genes integrated *de novo* prediction with homology and transcriptome data based evidence. Homology sequences from Pacific oyster (*Crassostrea gigas*), Molluscs (*Lottia gigantea*), Mosquito (*Anopheles gambiae*), Amphioxus (*Branchiostoma floridae*), Nematode (*Caenorhabditis elegans*), Ascidian (*Ciona intestinalis*), Fruit fly (*Drosophila melanogaster*), Leech (*Helobdella robusta*), Human (*Homo sapiens*), Octopuses (*Octopus bimaculoides*), Sea urchin (*Strongylocentrotus purpuratus*) were downloaded from Ensemble [21]. The protein sequences of homology species were aligned to the assembled genome with TBLASTn (e-value ≤ 10-5) [22] and predicted gene structures with GeneWise_2.4.1 (the parameter set as '-genesf') [23]. The transcriptome data were generated from adductor muscle, gill, hepatopancreas and mantle on Illumina

HiSeq4000 platform. And Tophat_2.1.1 (the parameter set as '--max-intron-length 500000 -m 2 --library-type fr-unstranded') [24] was utilized to map the transcriptome data onto our genome assembly and then Cufflinks_2.1.0 (the parameter set as '--multi-read-correct') [25] was employed to generated gene model according to the pair-end relationships and the overlap between aligned reads. The *de novo* prediction of genes was carried out with four programs: Augustus_3.0.3 (the parameter set as '-uniqueGeneId true --noInFrameStop=true --gff3 on –genemodel complete –strand both') [26], Genscan (the default parameter) [27], GlimmerHMM_3.0.2 (the parameter set as ' -f -g') [28] and SNAP (the default parameter) [29]. All evidences of gene model were integrated using EvidenceModeler_r2012-06-25 (EVM) [29]. Finally, we identified 26,256 protein-coding genes in the Peruvian scallop genome. A total of 26,513 genes were predicted through the *de novo* method, 19,394 genes were annotated by RNA transcripts or raw RNA reads, and 15,608 genes were supported by homolog evidence. The average transcript length, CDS length and intron length were calculated to be 10,534 bp, 1,418 bp and 1,505 bp respectively (Table 1).

*(2) Gene functional annotation*

Gene functions were predicted from the best BLASTP (e-value ≤ 10-5) hits in SwissProt databases [30]. Gene domain annotation was performed by searching the InterPro database [31]. All genes were aligned against Kyoto Encyclopedia of Genes and Genomes (KEGG) [32] to identify the best hits for pathways. Gene Ontology (GO) terms for genes were obtained from the corresponding InterPro entry [33]. Finally, among these annotated genes, 70.3% encoded proteins showed homology to proteins in the SwissProt database, 91.1% were identified in the non-redundant (Nr) database,

70.4% were identified in the KEGG database, 72.1% were identified in the InterPro, and a total of 92.1% could be mapped to the functional databases.

*(3) Non-coding RNA annotation*

The non-coding RNA genes, including miRNAs, rRNAs, snRNAs and tRNAs, were identified. The tRNAscan-SE 2.0 software with eukaryote parameters [34] was employed to predict tRNA genes. The miRNA and snRNA genes in the assembled genome were extracted by INFERNAL_1.1.2 software [35] against the Rfam database [36] with default parameters. Finally, 1,132 miRNAs, 1,664 tRNAs, 41 rRNAs and 220 snRNAs were discovered from the Peruvian scallop genome.

Global gene family classification

Protein-coding genes from the Peruvian scallop and other sequenced species, including Human (*H. sapiens*), Amphioxus (*B. floridae*), Fruit fly (*D. melanogaster*), Red flour beetle (*T. castaneum*), Nematode (*Caenorhabditis elegans*), brachiopod (*Lingula anatine*), *Helobdella robusta Capitella teleta*, *Octopus bimaculoides*, *Lottia gigantean*, mollusk (*Aplysia californica*), Pacific Abalone (*Haliotis discus*), Pacific oyster (*C. gigas*), pearl oyster (*Pinctada fucata*), Yesso scallop (*Patinopecten yessoensis*), and cold seep mussel (*Bathymodiolus platifrons*), Brown mussel (*Modiolus philippinarum*) were analyzed. All data were downloaded from Ensemble [21] or NCBI [37]. For each protein-coding gene with alternative splicing isoforms, we only kept the longest protein sequence as the representative.

Gene family analysis was based on the homolog of gene sequences in related species, which was initially implemented by the alignment of an "all against all" BLASTP (with a cutoff of 1e-7), and subsequently the alignments with high-scoring

segment pairs were conjoined for each gene pair by TreeFam_3.0 [38]. To identify homologous gene pairs, we required more than 30% coverage of the aligned regions in both homologous genes. Finally, homologous genes were clustered into gene families by OrthoMCL-5 [39] with the optimized parameter of '-inflation 1.5'. All protein-coding genes from the examined 18 genomes were employed to assign gene families. In total, the protein-coding genes were classified into 45,268 families and 108 strict single-copy orthologs (Figure 2).

Phylogenetic analysis

Evolutionary analysis was performed using these single-copy protein-coding genes from the examined 18 species. Amino acid and nucleotide sequences of the ortholog genes were aligned by the multiple alignment software MUSCLE with default parameters [40]. A total number of 108 single-copy ortholog alignments were concatenated into a super alignment matrix of 242,085 nucleotides. A Maximum Likelihood method (ML) deduced tree was inferred based on the matrix of nucleotide sequences using RAxML_v8.0.19 with default nucleotide substitution model-PROTGAMMAAUTO [41]. Clade support was assessed using bootstrapping algorithm in the RAxML package with 100 alignment replicates (Figure 3) [42]. The constructed phylogenetic tree (Figure 3) indicated that the Peruvian scallop and Yesso scallop are clustered closely first and then clustered with Oysters and Mussels, which is in consistent with their putative evolution relationships [43-46].

The estimation of divergence time

The species divergence times were inferred with MCMCTree included in PAML v4.7a [47] with the parameter set as 'burn-in=1,000, sample-number=1000,000,

sample-frequency=2', and evolutionary analysis was performed using single-copy protein-coding genes from the 18 examined species. Based on the phylogenetic tree (Figure 3), the molecular clock was calibrated based on the fossil records according to previous studies [48-51]. Finally, we estimated that the divergence between the Peruvian scallop and Yesso scallops happened at 113.6 Mya ago.

## Conclusion

In the present study, we reported the first whole genome sequencing, assembly and annotation of Peruvian scallop (*A. purpuratus*), an economically important bivalve in China. The assembled draft genome of 724.78 Mb accounts for 81.87% of the estimated genome size (885.29 Mb). A total of 26,256 protein-coding genes and 3.057 non-coding RNAs were predicted from the assembly. In the coming future, this generated genome assembly will provide solid support for deep biological studies. With availability of these genomic data, subsequent development of genetic markers for further genetic selection and molecular breeding of scallops could be realized. Our current genome data will also definitely facilitate the genetic evolutionary history analysis for the abundant scallops in the world.

## Availability of Data

Supporting raw data have been deposited in NCBI with the project accession PRJNA418203.


## Acknowledgements

This work was supported by National Natural Science Foundation of China (31572618 granted to C. Wang and 41676152 granted to X. Liu) and the earmarked

fund for Shandong Modern Agro-Industry Technology Research System (SDAIT-14) granted to C. Wang.

## Conflicts of interest

The authors declare that they have no competing interests.

## Author’s contributions

C.W., X.L. and C.L. designed the project. B.M. and G.L. collected the samples and prepared the quality control. C.L., C.W. and X.L. were involved in the data analysis. C.W., X.L., C.L. and Q.S. wrote the manuscript. All authors read and approved the final manuscript.

## Reference

1. Dall WH. The mollusca and branchiopoda. Report of dredging operation, Albatros' 1891. Bulletin Mollusca Comparative Zoology, 1909; 37: 147-294.
2. Gonzalez ML, Lopez DA, Perez MC, Riquelme VA, Uribe JM and Le PM. Growth and the scallop, *Argopecten purpuratu*s (Lamarck, 1819), in southern Chile. Aquaculture. 1999; 175 3–4:307-16.
3. Genética D, Morfológica Y, Dos E, Del P, Argopecten P, De P, et al. Genetic and morphological differentiation between two pectinid populations of *Argopecten purpuratus* from the northern Chilean coast. Estudios Oceanologicos. 2001; 1:51-60.
4. Disalvo LH, Alarcon E, Martinez E and Uribe E. Progress in mass culture of *Chlamys* (*Argopecten*) *purpurata* Lamarck (1819) with notes on its natural history. Revista Chilena de Historia Natural. 1984, 57:35-45.
5. Estabrooks SL. The possible role of telomeres in the short life span of the bay scallop, *Argopecten irradians irradians* (Lamarck 1819). Journal of Shellfish Research. 2007; 26 2:307-13.
6. Wang C, Liu B, Li J and Liu S. Inter-specific hybridization between *Argopecten purpuratus* and *Argopecten irradians irradians*. Marine Sciences. 2009; 33 10:84-75.

7. Wang C, Liu B, Li J, Liu S, Hu L, Fan X, et al. Introduction of the Peruvian scallop and its hybridization with the bay scallop in China. Aquaculture. 2011; 310 3–4:380-7.

8. Marçais G and Kingsford C. A fast, lock-free approach for efficient parallel counting of occurrences of k-mers. Bioinformatics. 2011; 27 6:764.

9. Kajitani R, Toshimoto K, Noguchi H, Toyoda A, Ogura Y, Okuno M, et al. Efficient de novo assembly of highly heterozygous genomes from whole-genome shotgun short reads. Genome Research. 2014; 24 8:1384-95.

10. Luo R, Liu B, Xie Y, Li Z, Huang W, Yuan J, et al. SOAPdenovo2: an empirically improved memory-efficient short-read *de novo* assembler. Gigascience. 2012; 1 1:18.

11. English AC, Richards S, Han Y, Wang M, Vee V, Qu J, et al. Mind the gap: upgrading genomes with Pacific Biosciences RS long-read sequencing technology. Plos One. 2012; 7 11:e47768.

12. Walker BJ, Abeel T, Shea T, Priest M, Abouelliel A, Sakthikumar S, et al. Pilon: an integrated tool for comprehensive microbial variant detection and genome assembly improvement. Plos One. 2014; 9 11:e112963.

13. Adey A, Kitzman JO, Burton JN, Daza R, Kumar A, Christiansen L, et al. In vitro, long-range sequence information for *de novo* genome assembly via transposase contiguity. Genome Research. 2014; 24 12:2041.

14. Li H and Durbin R. Fast and accurate short read alignment with Burrows-Wheeler transform. 2009; 25 14:1754-1760..

15. Parra G, Bradnam K and Korf I. CEGMA: a pipeline to accurately annotate core genes in eukaryotic genomes. Bioinformatics. 2007; 23 9:1061.

16. Simão FA, Waterhouse RM, Ioannidis P, Kriventseva EV and Zdobnov EM. BUSCO: assessing genome assembly and annotation completeness with single-copy orthologs. Bioinformatics. 2015; 31 19:3210.

17. Grundmann N, Demester L and Makalowski W. TEclass-a tool for automated classification of unknown eukaryotic transposable elements. Bioinformatics. 2009; 25 10:1329.

18. Jurka J, Kapitonov VV, Pavlicek A, Klonowski P, et al. Repbase Update, a database of eukaryotic repetitive elements. Cytogenetic & Genome Research. 2005 110:462-7.

19. Tarailograovac M and Chen N. Using RepeatMasker to identify repetitive elements in genomic sequences. Current protocols in bioinformatics. 2009; Chapter 4 Unit 4:Unit 4.10.

20. Benson G. Tandem repeats finder: a program to analyze DNA sequences. Nucleic Acids Research. 1999; 27 2:573-80.

21. Kersey PJ, Allen JE, Allot A, Barba M, Boddu S, Bolt BJ, et al. Ensembl Genomes 2018: an integrated omics infrastructure for non-vertebrate species. Nucleic Acids Res. 2017; doi:10.1093/nar/gkx1011.

22. Kent WJ. BLAT--the BLAST-like alignment tool. Genome Research. 2002; 12 4:656-64.

23. Birney E, Clamp M and Durbin R. GeneWise and Genomewise. Genome Research. 2004; 14 5:988.

24. Trapnell C, Pachter L and Salzberg SL. TopHat: discovering splice junctions with RNA-Seq. Bioinformatics. 2009; 25 9:1105-11.

25. Pertea G. Transcript assembly and quantification by RNA-Seq reveals unannotated transcripts and isoform switching during cell differentiation. 2010.

26. Stanke M and Waack S. Gene prediction with a hidden Markov model and a new intron submodel. Bioinformatics. 2003; 19 suppl_2:215-25.

27. Salamov AA and Solovyev VV. Ab initio gene finding in *Drosophila* genomic DNA. Genome Research. 2000; 10 4:516.

28. Majoros WH, Pertea M and Salzberg SL. TigrScan and GlimmerHMM: two open source ab initio eukaryotic gene-finders. Bioinformatics. 2004; 20 16:2878-9.

29. Korf I. Gene finding in novel genomes. Bmc Bioinformatics. 2004; 5 1:59.

30. Bairoch A and Apweiler R. The SWISS-PROT protein sequence database and its supplement TrEMBL in 2000. Nucleic Acids Research. 2000; 28 1:45-8.

31. Mulder N and Apweiler R. InterPro and InterProScan: tools for protein sequence classification and comparison. Methods in Molecular Biology. 2007; 396:59.

32. Kanehisa M and Goto S. KEGG: Kyoto Encyclopedia of genes and genomes. Nucleic Acids Research. 2000; 27 1:29-34.

33. Sherlock G. Gene Ontology: tool for the unification of biology. Canadian Institute of Food Science & Technology Journal. 2009; 22 4:415.

34. Lowe TM and Eddy SR. tRNAscan-SE: a program for improved detection of transfer RNA genes in genomic sequence. Nucleic Acids Research. 1997; 25 5:955-64.

35. Nawrocki EP, Kolbe DL and Eddy SR. Infernal 1.0: inference of RNA alignments. Bioinformatics. 2009; 25 10:1335.

36. Griffithsjones S, Moxon S, Marshall M, Khanna A, Eddy SR and Bateman A. Rfam: annotating non-coding RNAs in complete genomes. Nucleic Acids Research. 2005; 33 Database issue:121-4.

37. Sayers EW, Barrett T, Benson DA, Bryant SH, Canese K, Chetvernin V, et al. Database resources of the national center for biotechnology information. In: *Haptics Symposium* 2010, pp.199-205.

38. Ruan J, Li H, Chen Z, Coghlan A, Coin LJ, Guo Y, et al. TreeFam: 2008 Update. Nucleic Acids Research. 2008; 36 Database issue:D735-D40.

39. Li L, Stoeckert CJ and Roos DS. OrthoMCL: Identification of ortholog groups for eukaryotic genomes. Genome Research. 2003; 13 9:2178.

40. Edgar RC. MUSCLE: multiple sequence alignment with high accuracy and high throughput. Nucleic Acids Research. 2004; 32 5:1792-7.

41. Stamatakis A. RAxML Version 8: A tool for Phylogenetic Analysis and post-analysis of large phylogenies. Bioinformatics. 2014; 30 9:1312.

42. Stamatakis A, Ott M and Ludwig T. RAxML-OMP: An Efficient Program for Phylogenetic Inference on SMPs. In: *International Conference on Parallel Computing Technologies* 2005, pp.288-302.

43. Sun J, Zhang Y, Xu T, Mu H, Lan Y, Fields CJ, et al. Adaptation to deep-sea chemosynthetic environments as revealed by mussel genomes. Nature Ecology & Evolution. 2017;1 5:121.

44. Shi W, Zhang J, Jiao W, Ji L, Xun X, Yan S, et al. Scallop genome provides insights into evolution of bilaterian karyotype and development. Nature Ecology & Evolution. 2017; 1 5:120.

45. Kocot KM, Cannon JT, Todt C, Citarella MR, Kohn AB, Meyer A, et al. Phylogenomics reveals deep molluscan relationships. Nature. 2011; 477 7365:452.

46. Smith SA, Wilson NG, Goetz FE, Feehery C, Andrade SC, Rouse GW, et al. Resolving the evolutionary relationships of molluscs with phylogenomic tools. Nature. 2011; 480 7377:364-7.

47. Yang Z. PAML: a program package for phylogenetic analysis by maximum likelihood. Computer Applications in the Biosciences Cabios. 1997; 13 5:555.

48. Simakov O, Kawashima T, Marlétaz F, Jenkins J, Koyanagi R, Mitros T, et al. Hemichordate genomes and deuterostome origins. Nature. 2015; 527 7579:459-65.

49. Benton, M, Donoghue, P & Asher, R. Calibrating and constraining molecular clocks. in H, S B. & S Kumar (eds), *The timetree of Life.* Oxford University Press, 2009, pp. 35 - 86.

50. Mergl M, Massa D and Plauchut B. Devonian and carboniferous brachiopods and bivalves of the Djado sub-basin (North Niger, SW Libya). Journal of the Czech Geological Society. 2001; 46 3:169-88.

51. Erwin DH, Laflamme M, Tweedt SM, Sperling EA, Pisani D and Peterson KJ. The Cambrian conundrum: early divergence and later ecological success in the early history of animals. Science. 2011; 334 6059:1091-7.

51. Erwin DH, Laflamme M, Tweedt SM, Sperling EA, Pisani D and Peterson KJ. The Cambrian conundrum: early divergence and later ecological success in the early history of animals. Science. 2011;334 6059:1091-7.

Table 1. Summary of the Peruvian scallop genome assembly and annotation

| **Genome assembly** | **Parameter** |
|---|---|
| Contig N50 size (kb) | 80.11 |
| Scaffold N50 size (Mb) | 1.02 |
| Estimated genome size (Mb) | 885.29 |
| Assembled genome size (Mb) | 724.78 |
| Genome coverage (×) | 303.83 |
| The longest scaffold (bp) | 11,125,544 |
| **Genome annotation** | **Parameter** |
| Protein-coding gene number | 26,256 |
| Average transcript length (kb) | 10.53 |
| Average CDS length (bp) | 1,418.29 |
| Average intron length (bp) | 1,505.92 |
| Average exon length (bp) | 201.09 |
| Average exons per gene | 7.05 |

Table 2. The prediction of repeats elements in the Peruvian scallop genome.

| Type | Repeat Size (bp) | % of genome |
|---|---|---|
| TRF | 83,037,380 | 11.46 |
| RepeatMasker | 237,471,691 | 32.76 |
| RepeatProteinMask | 21,719,425 | 3.00 |
| Total | 294,496,811 | 40.63 |

**Figure 1** Picture of a representative Peruvian scallop in China.

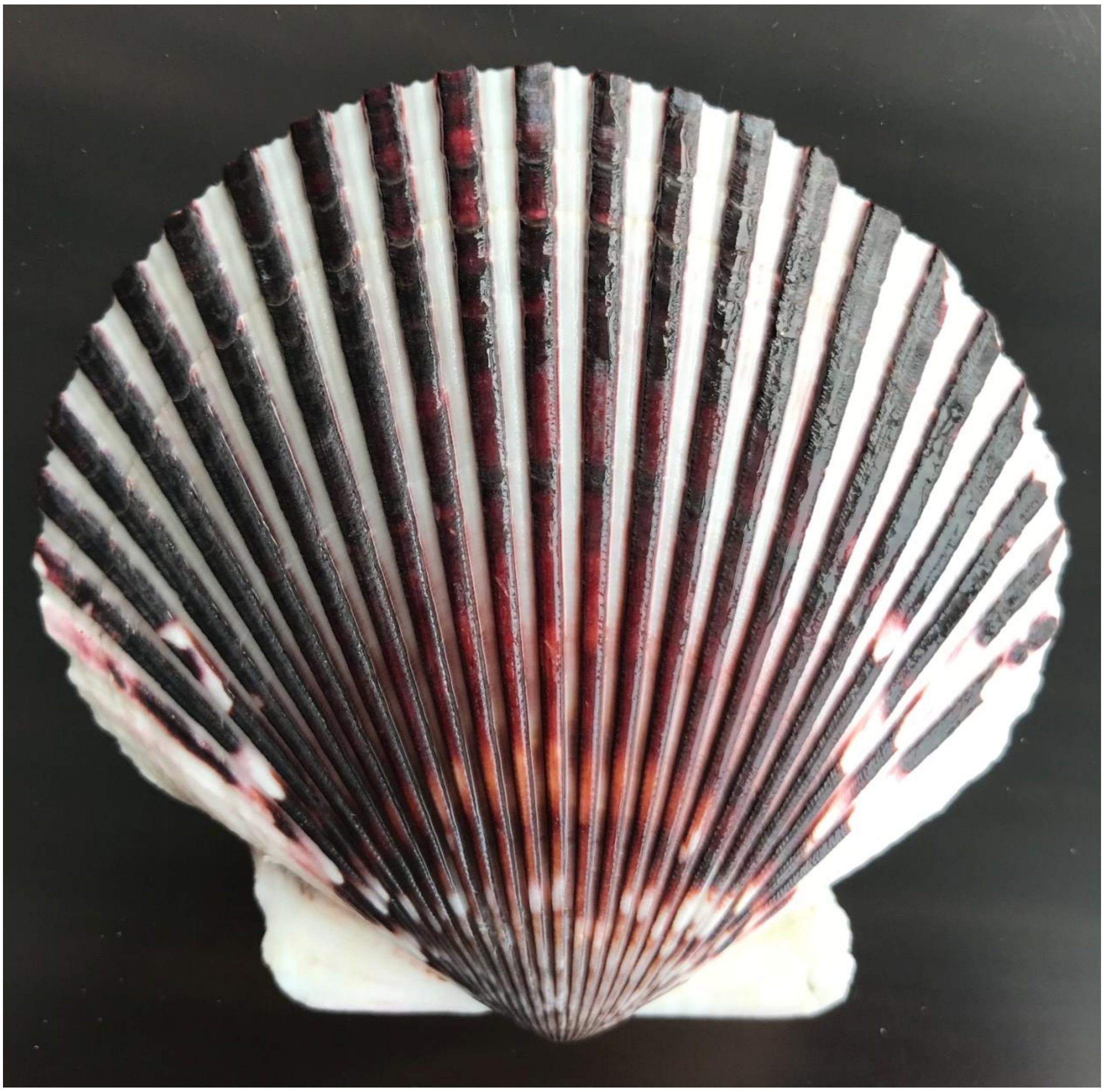

**Figure 2. Distribution of genes in different species.** Abbreviations: Aca, *Aplysia californica;* Apu, *Argopecten purpuratus;* Bfl, *Branchiostoma floridae;* Bpl, *Bathymodiolus platifrons;* Cel, *Caenorhabditis elegans;* Cgi, *Crassostrea gigas; Cte, Capitella teleta;* Dme, *Drosophila melanogaster;* Has, *Homo sapiens;* Hdi, *Haliotis discus;* Hro, *Helobdella robusta;* Lan, *Lingula anatine;* Lgi, *Lottia gigantean;* Mph, *Modiolus philippinarum;* Obi, *Octopus bimaculoides;* Pfu, *Pinctada fucata;* Tca, *Tribolium castaneum*.

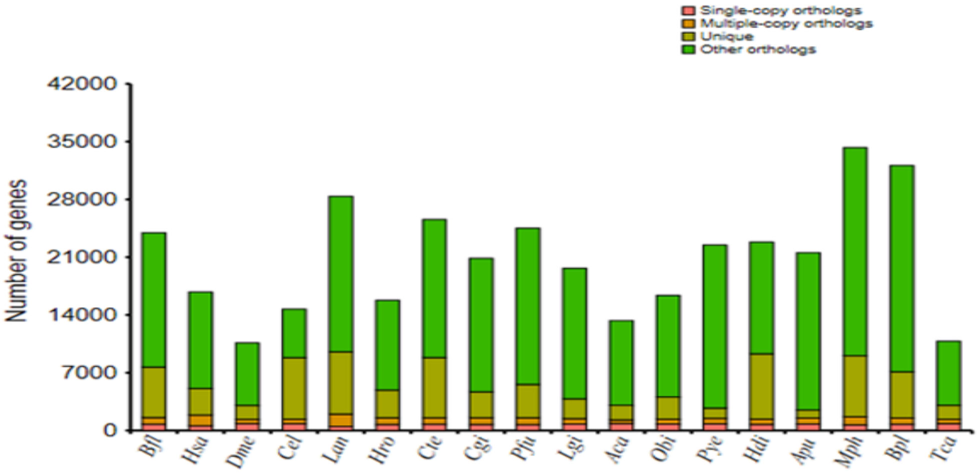

**Figure 3. Bootstrap support of phylogenetic tree.** A ML tree was constructed by RAxML based on 108 single-copy protein-coding genes of the related species. The total number of bootstrap was 100.

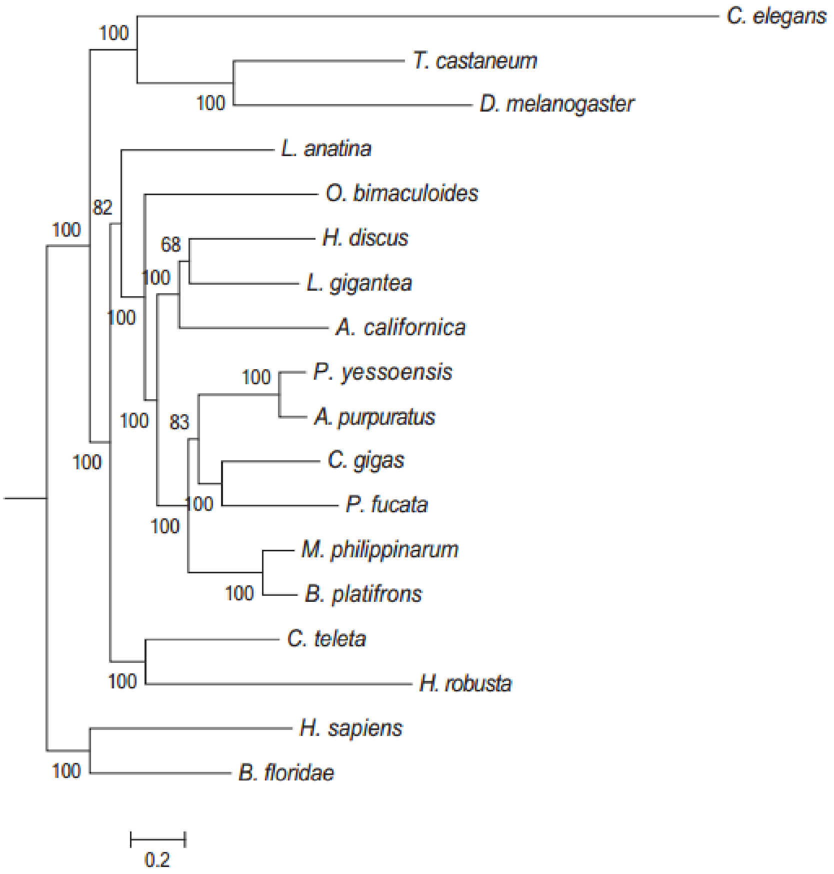

Click here to access/download
**Supplementary Material**
RESPONSE TO REVIEWERS.docx
