# Supplementary material for: Draft genome of the Peruvian scallop Argopecten purpuratus
Source: Gigascience. 2018 Apr 2;7(4):giy031. doi: 10.1093/gigascience/giy031 (PMC5905365; doi:10.1093/gigascience/giy031)

GigaScience

Draft genome of the Peruvian scallop Argopecten purpuratus

--Manuscript Draft--

Manuscript Number:	GIGA-D-17-00315R2
Full Title:	Draft genome of the Peruvian scallop Argopecten purpuratus
Article Type:	Data Note
Funding Information:	National Natural Science Foundation of China (31572618) — Dr. Chunde Wang<br>National Natural Science Foundation of China (41676152) — Dr. Xiao Liu<br>Fund for Shandong Modern Agro-Industry Technology Research System (SDAIT-14) — Dr. Chunde Wang
Abstract:	Background: The Peruvian scallop, Argopecten purpuratus, is mainly cultured in southern Chile and was introduced into China in last century. Unlike other Argopecten scallops, the Peruvian scallop normally has a long life span of up to 7-10 years. Therefore, researchers have been employing it to develop hybrid vigor. Here, we performed whole genome sequencing, assembly, and gene annotation of the Peruvian scallop, with an important aim to develop genomic resources for genetic breeding in scallops. Findings: A total of 463.19-Gb (Gigabase) raw DNA reads were sequenced. The draft genome assembly of 724.78 Mb was generated (accounting for 81.87% of the estimated genome size of 885.29 Mb), with a contig N50 size of 80.11 kb and a scaffold N50 size of 1.02 Mb. Meanwhile, the repeat sequences were calculated to reach 33.74% of the whole genome, and a total of 26,256 protein-coding genes and 3,057 non-coding RNAs were predicted from the assembly. Conclusion: We generated a high quality draft genome assembly of the Peruvian scallop, which will provide solid resource for further genetic breeding and evolutionary history analysis of this economically important scallop.
Corresponding Author:	Chunde Wang, Ph.D<br>Qingdao Agricultural University<br>CHINA
Corresponding Author Secondary Information:	
Corresponding Author's Institution:	Qingdao Agricultural University
Corresponding Author's Secondary Institution:	
First Author:	Chao Li, Ph.D
First Author Secondary Information:	
Order of Authors:	Chao Li, Ph.D<br>Xiao Liu, D.Sc<br>Bo Liu, D.Sc<br>Bin Ma, MSc<br>Fengqiao Liu<br>Guilong Liu, MSc<br>Qiong Shi, Ph.D<br>Chunde Wang, Ph.D
Order of Authors Secondary Information:	
Response to Reviewers:	Dear Editor,

Powered by Editorial Manager® and ProduXion Manager® from Aries Systems Corporation

	Thank you for the comments on the revised manuscript. We have corrected the manuscript thoroughly and have responded to the Reviewer's comments as attached below.<br>If you have more questions about the re-revised manuscript, please feel free to let us know. Thanks again for your kindly consideration of our paper.<br><br>Best regards,<br><br>Chunde and Chao<br><br>Reviewer reports:<br>Reviewer #2: I found all major questions by the reviewers were answered and sufficient information and data were added in the revised manuscript.<br>Now I would like to ask the authors to carefully correct some typos.<br><br>There are still uncorrected typos which the reviewers pointed out.<br>Please check the WHOLE sentences again and correct them.<br>Response: The manuscript has been revised carefully.<br><br>Lines 161-164 and Lines 207-212<br>Description of animal common names are inconsistent.<br>For example, "Molluscs", "Mosquito"... should be decapitalized.<br>Response: The names have been corrected.<br><br>Some are singular form and others are plural, which should be consistent.<br>i.e. "molluscs", "mosquitoes"... or "mollusca", "mosquito"...<br>Response: The names have been corrected!<br><br>In addition, in line 161 "Molluscs" while in line 210 "mollusk". They should be consistent.<br>Response: Corrected.<br><br>Line 210<br>"Lottia gigantean" should be "Lottia gigntea"<br>Response: Corrected.<br><br>Line 248<br>Mya stands for "million years ago." So "Mya ago" should be "Mya."<br>Response: Corrected.
**Additional Information:**	
**Question**	**Response**
Are you submitting this manuscript to a special series or article collection?	No
**Experimental design and statistics**<br><br>Full details of the experimental design and statistical methods used should be given in the Methods section, as detailed in our Minimum Standards Reporting Checklist. Information essential to interpreting the data presented should be made available in the figure legends.<br><br>Have you included all the information requested in your manuscript?	Yes

Powered by Editorial Manager® and ProduXion Manager® from Aries Systems Corporation

**Resources**<br><br>A description of all resources used, including antibodies, cell lines, animals and software tools, with enough information to allow them to be uniquely identified, should be included in the Methods section. Authors are strongly encouraged to cite Research Resource Identifiers (RRIDs) for antibodies, model organisms and tools, where possible.<br><br>Have you included the information requested as detailed in our Minimum Standards Reporting Checklist?	Yes
**Availability of data and materials**<br><br>All datasets and code on which the conclusions of the paper rely must be either included in your submission or deposited in publicly available repositories (where available and ethically appropriate), referencing such data using a unique identifier in the references and in the “Availability of Data and Materials” section of your manuscript.<br><br>Have you have met the above requirement as detailed in our Minimum Standards Reporting Checklist?	Yes

Manuscript

Click here to download Manuscript PeruvScallop-manuscript-final21-3-18.docx

Draft genome of the Peruvian scallop *Argopecten purpuratus*

Chao Li[1], Xiao Liu[2], Bo Liu[1], Bin Ma[3], Fengqiao Liu[1], Guilong Liu[1], Qiong Shi[4], Chunde Wang[1,*]

[1]Marine Science and Engineering College, Qingdao Agricultural University, Qingdao 266109, China

[2]Key Laboratory of Experimental Marine Biology, Institute of Oceanology, Chinese Academy of Sciences, Qingdao 266071, China

[3]Qingdao Oceanwide BioTech Co., Ltd., Qingdao 266101, China

[4]Shenzhen Key Lab of Marine Genomics, Guangdong Provincial Key Lab of Molecular Breeding in Marine Economic Animals, BGI Academy of Marine Sciences, BGI Marine, BGI, Shenzhen 518083, China

[*]Correspondence address. Professor Chunde Wang, Marine Science and Engineering College, Qingdao Agricultural University, Qingdao 266109, China (tel: +8613589227997; email: chundewang2007@163.com)

Email addresses of all authors:

Chao Li, leoochao@163.com

Xiao Liu, liuxiao@qdio.ac.cn

Bo Liu, liubomusic@126.com

Bin Ma, hereiammabin@163.com

Fengqiao Liu, liufengqiao2014@163.com

Guilong Liu, 969613442@qq.com

Qiong Shi, shiqiong@genomics.cn

Abstract

Background: The Peruvian scallop, *Argopecten purpuratus*, is mainly cultured in southern Chile and was introduced into China in the last century. Unlike other *Argopecten* scallops, the Peruvian scallop normally has a long life span of up to seven to ten years. Therefore, researchers have been employing it to develop hybrid vigor. Here, we performed whole genome sequencing, assembly, and gene annotation of the Peruvian scallop, with an important aim to develop genomic resources for genetic breeding in scallops. **Findings:** A total of 463.19-Gb (Gigabase) raw DNA reads were sequenced. A draft genome assembly of 724.78 Mb was generated (accounting for 81.87% of the estimated genome size of 885.29 Mb), with a contig N50 size of 80.11 kb and a scaffold N50 size of 1.02 Mb. Repeat sequences were calculated to reach 33.74% of the whole genome, and a total of 26,256 protein-coding genes and 3,057 non-coding RNAs were predicted from the assembly. **Conclusion:** We generated a high quality draft genome assembly of the Peruvian scallop, which will provide a solid resource for further genetic breeding and for the analysis of the evolutionary history of this economically important scallop.

Keywords: *Argopecten purpuratus*; Peruvian scallop; genome assembly; annotation; gene prediction; phylogenetic analysis

Data description

Introduction

The Peruvian scallop (*Argopecten purpuratus*), also known as the Chilean scallop, is a medium-sized bivalve with a wide distribution in Peru and Chile [1]. In Chile, the cultured scallops reach a commercial size of around 9 cm in shell height within 14-16 months [2]. It is a relatively stenothermic species as its natural habitat is largely under the influence of upwelling currents from Antarctica [3]. Unlike other *Argopecten* scallops, the Peruvian scallop normally has a long life span of up to 7-10 years [4, 5]. This scallop was introduced into China in the late 2000's and had played an important role in stock improvement of *Argopecten* scallops via inter-specific hybridization with bay scallops [6, 7].

Whole genome sequencing

Genomic DNA was extracted from adductor muscle sample of a single *A. purpuratus* (Figure 1), which was obtained from a local scallop farm in Laizhou, Shandong Province, China. A whole genome shotgun sequencing strategy was then applied. Briefly, six libraries with different insert length (250 bp, 450 bp, 2 kb, 5 kb, 10 kb, and 20 kb) were constructed according to the standard protocol provided by Illumina (San Diego, CA, USA). In detail, the DNA sample was randomly broken into fragments by covaris ultrasonic fragmentation apparatus. The library was prepared following end repair, adding sequence adaptor, purification, and PCR amplification. The mate-pair libraries (2 kb, 5 kb, 10 kb, and 20 kb) and paired-end libraries (250 bp,

450 bp) were all sequenced on Illumina HiSeq4000 platform with paired-end 150 bp. In addition, SMRTbell libraries were prepared using either 10-kb or 20-kb preparation protocols. Briefly, the DNA sample was sheared by Diagenode Megaruptor2 (the Kingdom of Belgium), the SMRTbell library was produced by ligating universal hairpin adapters onto double-stranded DNA fragments. Adapter dimers were efficiently removed using PacBio's MagBead kit. The final step of the protocol was to remove failed ligation products through the use of exonucleases. After the exonuclease and AMPure PB purification steps, sequencing primer was annealed to the SMRTbell templates, followed by binding of the sequence polymerase to the annealed templates. Subsequent sequencing was performed on PacBio Sequel instrument with Sequel™ Sequencing Kit 1.2.1 (Pacific Biosciences of California，USA). Finally, the 10X Genomics library was constructed and sequenced with paired-end 150 bp on the Illumina Hiseq platform. The Chromium™ Genome Solution（10X Genomics，USA） massively partitions and molecularly barcodes DNA using microfluidics, producing sequencing-ready libraries with >1,000,000 unique barcodes. In total, 463.19 gigabases (Gb) raw reads were generated, including 75.72, 70.22, 19.21, 45.71, 28.34, 11.78, 18.01 and 194.20 Gb from the 250-bp, 450-bp, 2-kb, 5-kb, 10-kb, 20-kb libraries, Pacbio sequencing library, and 10X Genomics library, respectively. The raw reads were trimmed before being used for subsequent genome assembly. For Illumina HiSeq sequencing, the adaptor sequences, the reads containing more than 10% ambiguous nucleotides, as well as the reads containing more than 20% low quality nucleotides (quality score less than 5) were all removed. For PacBio sequencing, the generated polymerase reads were firstly broken at the adaptor positions and the subreads were generated following removing the adaptor sequences. The subreads were then filtered by minimum length = 50.

Estimation of the genome size and sequencing coverage

The 17-mer frequency distribution analysis [8] was performed on the remaining clean reads to estimate the genome size of the Peruvian scallop using the following formula: genome size = k-mer number / peak depth. Based on a total number of 6.22 10^{10} k-mers and a peak k-mer depth of 69, the estimated genome size was calculated to be 885.29 Mb (Table 1) and the estimated repeat sequencing ratio was 33.74%.

De novo genome assembly and quality assessment of *A. purpuratus* genome

All the pair-end Illumina reads were first assembled into scaffolds using Platanus_v1.2.4 (Platanus, RRID:SCR_015531) [9], and the gaps were then filled by GapCloser_v1.12-r6 (GapCloser, RRID:SCR_015026) [10]. Subsequently, the Pacbio data were used for additional gap filling by PBJelly_v14.1 (PBJelly, RRID:SCR_012091) with default parameters [11], and then all the Illumina reads were employed to correct the genome assembly by Pilon_v1.18 (Pilon, RRID:SCR_014731) for two rounds [12]. After that, the 10X linked-reads were used to link scaffolds by fragScaff_140324.1 [13]. Particularly, in order to solve the issue of heterozygosity, in our assembly process, we chose 19-kmer to draw k-mer distribution histogram, and classified all the kmers into homozygous kmer and heterozygous kmer according to the coverage depth. Secondly, we utilized 45-kmer to construct the de bruijn figure and combine the bubbles for heterozygous sites, according to the sequences with longer length and deeper coverage depth. Then the pair-end information was used to determine the connection between the heterozygous parts, and filter the contigs lacking support. Finally, the heterozygous contigs and homozygous contigs were distinguished based on contig coverage depth. After

assembly, the reads from short insert length libraries were mapped onto the assembled genome. And only one peak was observed in the sequencing depth distribution analysis with the average sequencing depth of 148.2×, which is consistent with the sequencing depth, indicating high quality of the assembled scallop genome. Finally, a draft genome of 724.78 Mb was assembled (accounting for 81.87% of the estimated genome size of 885.29 Mb), with a contig N50 size of 80.11 kb and scaffold N50 size of 1.02 Mb (Table 1).

With this initial assembly, we mapped the short insert library reads onto the assembled genome using BWA_0.6.2 (BWA, RRID:SCR_010910) software [14] to calculate the mapping ratio and assess the assembly integrity. In summary, 91.05% of the short reads were mapped onto the assembled genome with a coverage of 89.40%, indicating high reliability of genome assembly. CEGMA_v2.5 (Core Eukaryotic Genes Mapping Approach; CEGMA, RRID:SCR_015055) defines a set of conserved protein families that occur in a wide range of eukaryotes, and presents a mapping procedure to accurately identify their exon-intron structures in a novel genomic sequence [15]. A protein is classified as complete if the alignment of the predicted protein to the HMM profile represents at least 70% of the original KOG domain, or otherwise classified as partial. Through mapping to the 248 core eukaryotic genes, a total of 222 genes (89.52%) were identified. BUSCO_v3 (Benchmarking Universal Single-Copy Orthologs; BUSCO, RRID:SCR_015008) provides quantitative measures for the assessment of genome assembly completeness, based on evolutionarily-informed expectations of gene content from near-universal single-copy orthologs [16]. We confirmed that 89% of the 843 single-copy genes were identified, indicating a good integrity of the genome assembly.

Repeat sequence analysis of the genome assembly

We searched transposable elements (TEs) in the assembled genome through *ab-initio* and homology-based methods. For the first method, we applied RepeatModeler_1.0.4 (RepeatModeler, RRID:SCR_015027) [17] (the parameter set as '--engine_db wublast') to build a specific repeat database. For the second method, we employed known repeat library (Repbase) [18] to identify repeats with RepeatMasker_open-4.0 [19] (the parameter set as '-a -nolow -no_is -norna -parallel 3 -e wublast --pvalue 0.0001') and RepeatProteinMask (the parameter set as '-noLowSimple -pvalue 0.0001 -engine wublast') [19]. Tandem repeats finder_4.04 (TRF) was used to find tandem repeats with the parameters setting as 'Match = 2, Mismatching penalty = 7, Delta = 7, PM = 80, PI = 10, Minscore = 50, MaxPeriod = 2,000' [20]. Finally, we summarized that the total repeat sequences are 294,496,811 bp, accounting for 40.63% of the assembled genome, and including 11.46% of tandem repeats, which is consistent with our above-mentioned estimation (Table 2).

Gene annotation

(1) Annotation of protein coding genes

The annotation strategy for protein-coding genes integrated *de novo* prediction with homology and transcriptome data based evidence. Homology sequences from African malaria mosquito (*Anopheles gambiae*), Ascidian (*Ciona intestinalis*), Florida lancelet (*Branchiostoma floridae*), Fruit fly (*Drosophila melanogaster*), Human (*Homo sapiens*), Leech (*Helobdella robusta*), Nematode (*Caenorhabditis elegans*), Octopuse (*Octopus bimaculoides*), Owl limpet (*Lottia gigantea*), Pacific oyster (*Crassostrea gigas*), Sea urchin (*Strongylocentrotus purpuratus*) were downloaded from Ensemble [21]. The protein sequences of homology species were aligned to the

assembled genome with TBLASTn (e-value ≤ 10^{-5}) [22], and gene structures were predicted with GeneWise_2.4.1 (GeneWise, RRID:SCR_015054) (the parameter set as '-genesf') [23]. The transcriptome data were generated from adductor muscle, hepatopancreas and mantle on Illumina HiSeq4000 platform. And Tophat_2.1.1 (the parameter set as '--max-intron-length 500000 -m 2 --library-type fr-unstranded') [24] was utilized to map the transcriptome data onto genome assembly and then Cufflinks_2.1.0 (Cufflinks, RRID:SCR_014597), the parameter set as '--multi-read-correct', [25] was employed to generate gene model according to the pair-end relationships and the overlap between aligned reads. The *de novo* prediction of genes was carried out with four programs: Augustus_3.0.3 (Augustus: Gene Prediction, RRID:SCR_008417), the parameter set as '-uniqueGeneId true --noInFrameStop=true --gff3 on –genemodel complete –strand both' [26]; GENSCAN (GENSCAN, RRID:SCR_012902), with default parameter [27]; GlimmerHMM_3.0.2 (GlimmerHMM, RRID:SCR_002654), the parameter set as ' -f -g' [28]; and SNAP (the default parameter) [29]. All evidences of gene model were integrated using EvidenceModeler_r2012-06-25 (EVM) [29]. Finally, we identified 26,256 protein-coding genes in Peruvian scallop genome. In detail, a total of 26,513 genes were predicted through the *de novo* method, 19,394 genes were annotated by RNA transcripts or raw RNA reads, and 15,608 genes were supported by homolog evidences. The average transcript length, CDS length and intron length were 10,534 bp, 1,418 bp and 1,505 bp, respectively (Table 1).

(2) Gene functional annotation

Gene functions were predicted from the best BLASTP (e-value ≤ 10^{-5}) hits in SwissProt databases [30]. Gene domain annotation was performed by searching the

InterPro (InterPro, RRID:SCR_006695) database [31]. All genes were aligned against Kyoto Encyclopedia of Genes and Genomes (KEGG, RRID:SCR_012773) [32] to identify the best hits for pathways. Gene Ontology (GO) terms for genes were obtained from the corresponding InterPro entry [33]. Finally, among these annotated genes, 70.3% of encoded proteins showed homology to proteins in the SwissProt database, 91.1% were identified in the non-redundant (Nr) database, 70.4% were identified in the KEGG database, 72.1% were identified in the InterPro, and a total of 92.1% could be mapped onto the functional databases.

(3) Non-coding RNA annotation

The non-coding RNA genes, including miRNAs, rRNAs, snRNAs and tRNAs, were identified. The tRNAscan-SE_2.0 (tRNAscan-SE, RRID:SCR_010835) software with eukaryote parameters [34] was employed to predict tRNA genes. The miRNA and snRNA genes in the assembled genome were extracted by INFERNAL_1.1.2 software [35] against the Rfam (Rfam, RRID:SCR_007891) database [36] with default parameters. Finally, 1,132 miRNAs, 1,664 tRNAs, 41 rRNAs and 220 snRNAs were discovered from the Peruvian scallop genome.

Global gene family classification

Protein-coding genes from the Peruvian scallop and other sequenced species, including Brachiopod (*Lingula anatina*), Brown mussel (*Modiolus philippinarum*), California sea hare (*Aplysia californica*), cold seep mussel (*Bathymodiolus platifrons*), Florida lancelet (*B. floridae*), Fruit fly (*D. melanogaster*), Human (*H. sapiens*), Leech (*H. robusta*, *Capitella teleta*), Nematode (*C. elegans*), Octopus (*O. bimaculoides*), Owl limpet (*L. gigantea*), Pacific abalone (*Haliotis discus*), Pacific oyster (*C. gigas*),

Pearl oyster (*Pinctada fucata*), Red flour beetle (*Tribolium castaneum*), and Yesso scallop (*Patinopecten yessoensis*) were analyzed. All data were downloaded from Ensemble [21] or NCBI [37]. For each protein-coding gene with alternative splicing isoforms, only the longest protein sequence was kept as the representative.

Gene family analysis based on the homolog of gene sequences in related species was initially implemented by the alignment of an "all against all" BLASTP (with a cutoff of 1e-7) and subsequently followed by alignments with high-scoring segment pairs conjoined for each gene pair by TreeFam_3.0 [38]. To identify homologous gene pairs, we required more than 30% coverage of the aligned regions in both homologous genes. Finally, homologous genes were clustered into gene families by OrthoMCL-5 (OrthoMCL DB: Ortholog Groups of Protein Sequences, RRID:SCR_007839) [39] with the optimized parameter of '-inflation 1.5'. All protein-coding genes from the examined 18 genomes were employed to assign gene families. In total, the protein-coding genes were classified into 45,268 families and 108 strict single-copy orthologs (Figure 2).

Phylogenetic analysis

Evolutionary analysis was performed using these single-copy protein-coding genes from the examined 18 species. Amino acid and nucleotide sequences of the ortholog genes were aligned by the multiple alignment software MUSCLE (MUSCLE, RRID:SCR_011812) with default parameters [40]. A total number of 108 single-copy ortholog alignments were concatenated into a super alignment matrix of 242,085 nucleotides. A Maximum Likelihood method (ML) deduced tree was inferred based on the matrix of nucleotide sequences using RAxML_v8.0.19 (RAxML, RRID:SCR_006086) with default nucleotide substitution

model-PROTGAMMAAUTO [41]. Clade support was assessed using bootstrapping algorithm in the RAxML package with 100 alignment replicates (Figure 3) [42]. The constructed phylogenetic tree (Figure 3) indicated that the Peruvian scallop and Yesso scallop were clustered closely first and then clustered with oysters and mussels, which is in consistent with their putative evolution relationships [43-46].

The estimation of divergence time

The species divergence times were inferred with MCMCTree included in PAML v4.7a (PAML, RRID:SCR_014932) [47] with the parameter set as 'burn-in=1,000, sample-number=1000,000, sample-frequency=2', and evolutionary analysis was performed using single-copy protein-coding genes from the 18 examined species. Based on the phylogenetic tree (Figure 3), the molecular clock was calibrated based on the fossil records according to previous studies [48-51]. Finally, we estimated that the divergence between the Peruvian scallop and Yesso scallop happened at 113.6 Mya.

Conclusion

In the present study, we report the first whole genome sequencing, assembly and annotation of the Peruvian scallop (*A. purpuratus*), an economically important bivalve in Chile, Peru and China. The assembled draft genome of 724.78 Mb accounts for 81.87% of the estimated genome size (885.29 Mb). A total of 26,256 protein-coding genes and 3,057 non-coding RNAs were predicted from the genome assembly. This genome assembly will provide solid support for in-depth biological studies. With the availability of these genomic data, subsequent development of genetic markers for further genetic selection and molecular breeding of scallops could be realized. The

current genome data will also facilitate genetic analyses of the evolutionary history of the abundant scallops in the world.

Availability of Data

Supporting data are available in the *GigaScience* database [52]. Raw data have been deposited in NCBI with the project accession PRJNA418203. BioSample accessions: SAMN08022140 (genome); SAMN08731415 (transcriptome; muscle) SAMN08731411 (transcriptome; mantle); SAMN08731410 (transcriptome; hepatopancreas).

Acknowledgements

This work was supported by National Natural Science Foundation of China (31572618 granted to C. Wang and 41676152 granted to X. Liu) and the earmarked fund for Shandong Modern Agro-Industry Technology Research System (SDAIT-14) granted to C. Wang.

Conflicts of interest

The authors declare that they have no competing interests.

Author's contributions

C.W., X.L. and C.L. designed the project. B.M., F.L. and G.L. collected the samples and prepared the quality control. C.L., C.W. and X.L. were involved in the data analysis. C.W., X.L., C.L. and Q.S. wrote the manuscript. All authors read and approved the final manuscript.

Reference

1. Dall WH. The mollusca and branchiopoda. Report of dredging operation, Albatros' 1891. Bulletin Mollusca Comparative Zoology, 1909; 37: 147-294.
2. Gonzalez ML, Lopez DA, Perez MC, Riquelme VA, Uribe JM and Le PM. Growth and the scallop, *Argopecten purpuratu*s (Lamarck, 1819), in southern Chile. Aquaculture. 1999; 175 3–4:307-16.
3. Genética D, Morfológica Y, Dos E, Del P, Argopecten P, De P, et al. Genetic and morphological differentiation between two pectinid populations of *Argopecten purpuratus* from the northern Chilean coast. Estudios Oceanologicos. 2001; 1:51-60.
4. Disalvo LH, Alarcon E, Martinez E and Uribe E. Progress in mass culture of *Chlamys* (*Argopecten*) *purpurata* Lamarck (1819) with notes on its natural history. Revista Chilena de Historia Natural. 1984, 57:35-45.
5. Estabrooks SL. The possible role of telomeres in the short life span of the bay scallop, *Argopecten irradians irradians* (Lamarck 1819). Journal of Shellfish Research. 2007; 26 2:307-13.
6. Wang C, Liu B, Li J and Liu S. Inter-specific hybridization between *Argopecten purpuratus* and *Argopecten irradians irradians*. Marine Sciences. 2009; 33 10:84-75.
7. Wang C, Liu B, Li J, Liu S, Hu L, Fan X, et al. Introduction of the Peruvian scallop and its hybridization with the bay scallop in China. Aquaculture. 2011; 310 3–4:380-7.
8. Marçais G and Kingsford C. A fast, lock-free approach for efficient parallel counting of occurrences of k-mers. Bioinformatics. 2011; 27 6:764.
9. Kajitani R, Toshimoto K, Noguchi H, Toyoda A, Ogura Y, Okuno M, et al. Efficient de novo assembly of highly heterozygous genomes from whole-genome shotgun short reads. Genome Research. 2014; 24 8:1384-95.
10. Luo R, Liu B, Xie Y, Li Z, Huang W, Yuan J, et al. SOAPdenovo2: an empirically improved memory-efficient short-read *de novo* assembler. Gigascience. 2012; 1 1:18.
11. English AC, Richards S, Han Y, Wang M, Vee V, Qu J, et al. Mind the gap: upgrading genomes with Pacific Biosciences RS long-read sequencing technology. Plos One. 2012; 7 11:e47768.
12. Walker BJ, Abeel T, Shea T, Priest M, Abouelliel A, Sakthikumar S, et al. Pilon: an integrated tool for comprehensive microbial variant detection and genome assembly improvement. Plos One. 2014; 9 11:e112963.
13. Adey A, Kitzman JO, Burton JN, Daza R, Kumar A, Christiansen L, et al. In vitro, long-range sequence information for *de novo* genome assembly via transposase contiguity. Genome Research. 2014; 24 12:2041.
14. Li H and Durbin R. Fast and accurate short read alignment with Burrows-Wheeler transform. 2009; 25 14:1754-1760..
15. Parra G, Bradnam K and Korf I. CEGMA: a pipeline to accurately annotate core genes in eukaryotic genomes. Bioinformatics. 2007; 23 9:1061.

16. Simão FA, Waterhouse RM, Ioannidis P, Kriventseva EV and Zdobnov EM. BUSCO: assessing genome assembly and annotation completeness with single-copy orthologs. Bioinformatics. 2015; 31 19:3210.
17. Grundmann N, Demester L and Makalowski W. TEclass-a tool for automated classification of unknown eukaryotic transposable elements. Bioinformatics. 2009; 25 10:1329.
18. Jurka J, Kapitonov VV, Pavlicek A, Klonowski P, et al. Repbase Update, a database of eukaryotic repetitive elements. Cytogenetic & Genome Research. 2005 110:462-7.
19. Tarailograovac M and Chen N. Using RepeatMasker to identify repetitive elements in genomic sequences. Current protocols in bioinformatics. 2009; Chapter 4 Unit 4:Unit 4.10.
20. Benson G. Tandem repeats finder: a program to analyze DNA sequences. Nucleic Acids Research. 1999; 27 2:573-80.
21. Kersey PJ, Allen JE, Allot A, Barba M, Boddu S, Bolt BJ, et al. Ensembl Genomes 2018: an integrated omics infrastructure for non-vertebrate species. Nucleic Acids Res. 2017; doi:10.1093/nar/gkx1011.
22. Kent WJ. BLAT--the BLAST-like alignment tool. Genome Research. 2002; 12 4:656-64.
23. Birney E, Clamp M and Durbin R. GeneWise and Genomewise. Genome Research. 2004; 14 5:988.
24. Trapnell C, Pachter L and Salzberg SL. TopHat: discovering splice junctions with RNA-Seq. Bioinformatics. 2009; 25 9:1105-11.
25. Pertea G. Transcript assembly and quantification by RNA-Seq reveals unannotated transcripts and isoform switching during cell differentiation. 2010.
26. Stanke M and Waack S. Gene prediction with a hidden Markov model and a new intron submodel. Bioinformatics. 2003; 19 suppl_2:215-25.
27. Salamov AA and Solovyev VV. Ab initio gene finding in *Drosophila* genomic DNA. Genome Research. 2000; 10 4:516.
28. Majoros WH, Pertea M and Salzberg SL. TigrScan and GlimmerHMM: two open source ab initio eukaryotic gene-finders. Bioinformatics. 2004; 20 16:2878-9.
29. Korf I. Gene finding in novel genomes. Bmc Bioinformatics. 2004; 5 1:59.
30. Bairoch A and Apweiler R. The SWISS-PROT protein sequence database and its supplement TrEMBL in 2000. Nucleic Acids Research. 2000; 28 1:45-8.
31. Mulder N and Apweiler R. InterPro and InterProScan: tools for protein sequence classification and comparison. Methods in Molecular Biology. 2007; 396:59.
32. Kanehisa M and Goto S. KEGG: Kyoto Encyclopedia of genes and genomes. Nucleic Acids Research. 2000; 27 1:29-34.

33. Sherlock G. Gene Ontology: tool for the unification of biology. Canadian Institute of Food Science & Technology Journal. 2009; 22 4:415.

34. Lowe TM and Eddy SR. tRNAscan-SE: a program for improved detection of transfer RNA genes in genomic sequence. Nucleic Acids Research. 1997; 25 5:955-64.

35. Nawrocki EP, Kolbe DL and Eddy SR. Infernal 1.0: inference of RNA alignments. Bioinformatics. 2009; 25 10:1335.

36. Griffithsjones S, Moxon S, Marshall M, Khanna A, Eddy SR and Bateman A. Rfam: annotating non-coding RNAs in complete genomes. Nucleic Acids Research. 2005; 33 Database issue:121-4.

37. Sayers EW, Barrett T, Benson DA, Bryant SH, Canese K, Chetvernin V, et al. Database resources of the national center for biotechnology information. In: *Haptics Symposium* 2010, pp.199-205.

38. Ruan J, Li H, Chen Z, Coghlan A, Coin LJ, Guo Y, et al. TreeFam: 2008 Update. Nucleic Acids Research. 2008; 36 Database issue:D735-D40.

39. Li L, Stoeckert CJ and Roos DS. OrthoMCL: Identification of ortholog groups for eukaryotic genomes. Genome Research. 2003; 13 9:2178.

40. Edgar RC. MUSCLE: multiple sequence alignment with high accuracy and high throughput. Nucleic Acids Research. 2004; 32 5:1792-7.

41. Stamatakis A. RAxML Version 8: A tool for Phylogenetic Analysis and post-analysis of large phylogenies. Bioinformatics. 2014; 30 9:1312.

42. Stamatakis A, Ott M and Ludwig T. RAxML-OMP: An Efficient Program for Phylogenetic Inference on SMPs. In: *International Conference on Parallel Computing Technologies* 2005, pp.288-302.

43. Sun J, Zhang Y, Xu T, Mu H, Lan Y, Fields CJ, et al. Adaptation to deep-sea chemosynthetic environments as revealed by mussel genomes. Nature Ecology & Evolution. 2017;1 5:121.

44. Shi W, Zhang J, Jiao W, Ji L, Xun X, Yan S, et al. Scallop genome provides insights into evolution of bilaterian karyotype and development. Nature Ecology & Evolution. 2017; 1 5:120.

45. Kocot KM, Cannon JT, Todt C, Citarella MR, Kohn AB, Meyer A, et al. Phylogenomics reveals deep molluscan relationships. Nature. 2011; 477 7365:452.

46. Smith SA, Wilson NG, Goetz FE, Feehery C, Andrade SC, Rouse GW, et al. Resolving the evolutionary relationships of molluscs with phylogenomic tools. Nature. 2011; 480 7377:364-7.

47. Yang Z. PAML: a program package for phylogenetic analysis by maximum likelihood. Computer Applications in the Biosciences Cabios. 1997; 13 5:555.

48. Simakov O, Kawashima T, Marlétaz F, Jenkins J, Koyanagi R, Mitros T, et al. Hemichordate genomes and deuterostome origins. Nature. 2015; 527 7579:459-65.

49. Benton, M, Donoghue, P & Asher, R. Calibrating and constraining molecular clocks. in H, S B. & S Kumar (eds), *The timetree of Life*. Oxford University Press, 2009, pp. 35 - 86.

50. Mergl M, Massa D and Plauchut B. Devonian and carboniferous brachiopods and bivalves of the Djado sub-basin (North Niger, SW Libya). Journal of the Czech Geological Society. 2001; 46 3:169-88.

51. Erwin DH, Laflamme M, Tweedt SM, Sperling EA, Pisani D and Peterson KJ. The Cambrian conundrum: early divergence and later ecological success in the early history of animals. Science. 2011; 334 6059:1091-7.

51. Erwin DH, Laflamme M, Tweedt SM, Sperling EA, Pisani D and Peterson KJ. The Cambrian conundrum: early divergence and later ecological success in the early history of animals. Science. 2011;334 6059:1091-7.

52. Li C, Liu X, Liu B, Ma B, Liu F, Liu G, Shi Q, Wang C. Supporting data for “Draft genome of the Peruvian scallop *Argopecten purpuratus*.” GigaScience Database 2018. http://dx.doi.org/10.5524/100419

Table 1. Summary of the Peruvian scallop genome assembly and annotation.

**Genome assembly**	**Parameter**
Contig N50 size (kb)	80.11
Scaffold N50 size (Mb)	1.02
Estimated genome size (Mb)	885.29
Assembled genome size (Mb)	724.78
Genome coverage ()	303.83
The longest scaffold (bp)	11,125,544
**Genome annotation**	**Parameter**
Protein-coding gene number	26,256
Average transcript length (kb)	10.53
Average CDS length (bp)	1,418.29
Average intron length (bp)	1,505.92
Average exon length (bp)	201.09
Average exons per gene	7.05

Table 2. The prediction of repeat elements in the Peruvian scallop genome.

**Type**	**Repeat Size (bp)**	**% of genome**
TRF	83,037,380	11.46
RepeatMasker	237,471,691	32.76
RepeatProteinMask	21,719,425	3.00
Total	294,496,811	40.63

Figure 1 Picture of a representative Peruvian scallop in China.

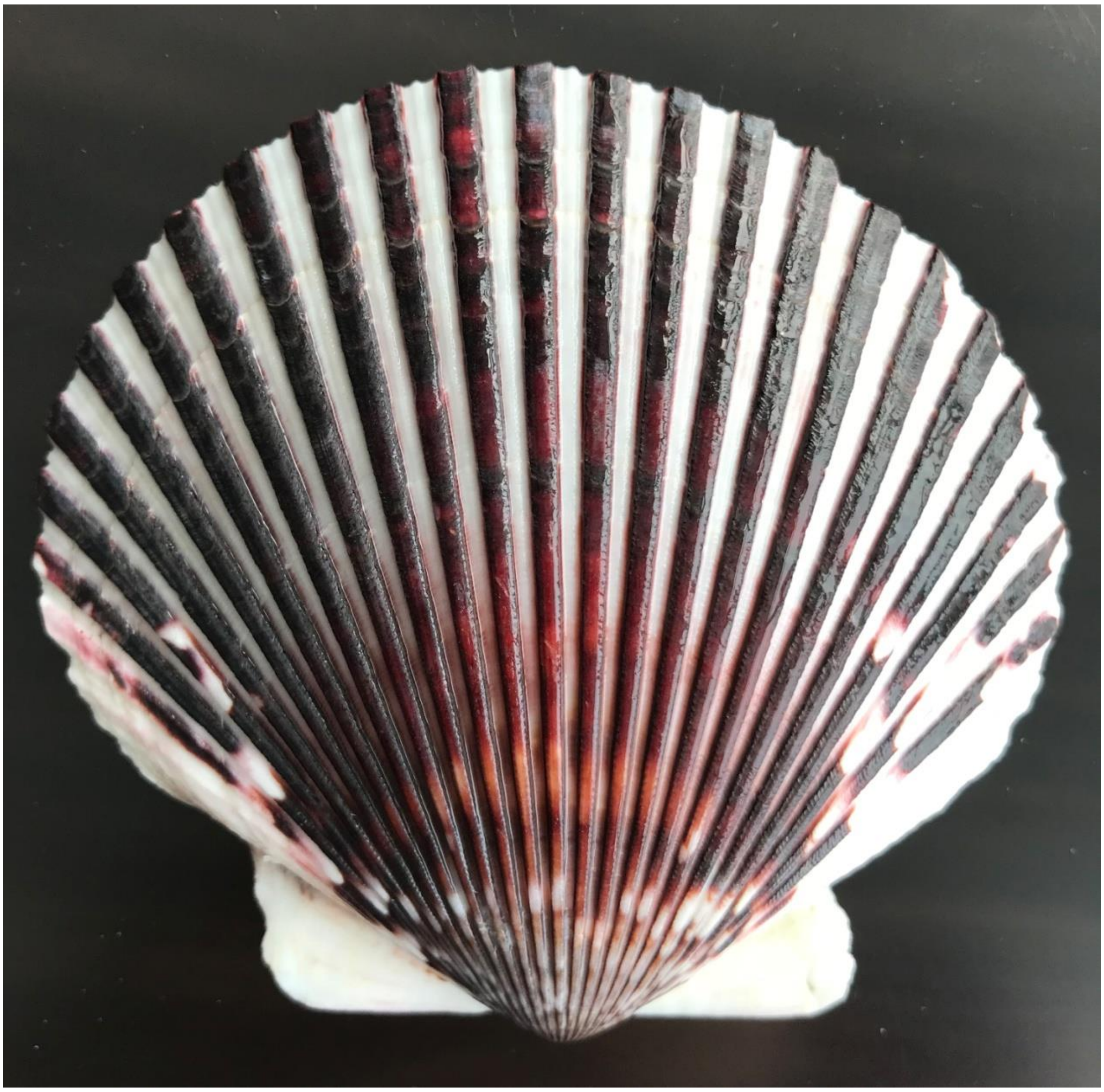

Figure 2. Distribution of genes in different species. Abbreviations: Aca, *Aplysia californica;* Apu, *Argopecten purpuratus;* Bfl, *Branchiostoma floridae;* Bpl, *Bathymodiolus platifrons;* Cel, *Caenorhabditis elegans;* Cgi, *Crassostrea gigas; Cte, Capitella teleta;* Dme, *Drosophila melanogaster;* Hsa, *Homo sapiens;* Hdi, *Haliotis discus;* Hro, *Helobdella robusta;* Lan, *Lingula anatina;* Lgi, *Lottia gigantea;* Mph, *Modiolus philippinarum;* Obi, *Octopus bimaculoides;* Pfu, *Pinctada fucata;* Pye, *Patinopecten yessoensis*; Tca, *Tribolium castaneum*.

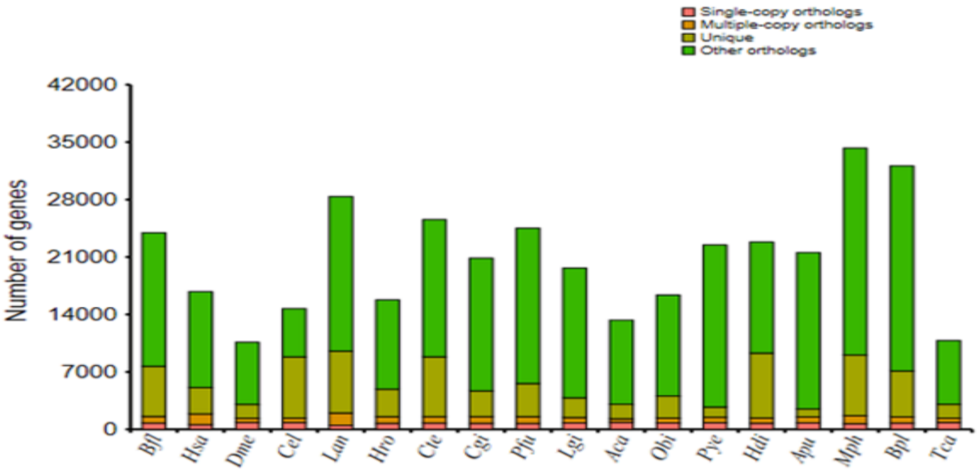

Figure 3. Bootstrap support of phylogenetic tree. A ML tree was constructed by RAxML based on 108 single-copy protein-coding genes of the related species. The total number of bootstrap was 100.

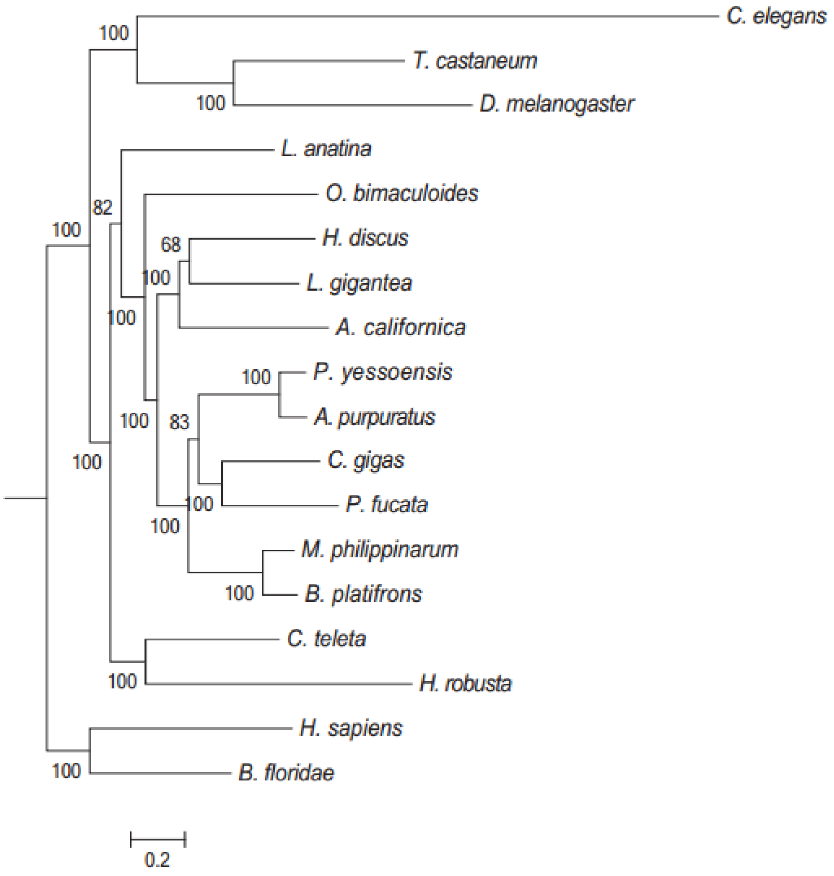

Figure

Click here to download Figure PSFig1.jpg

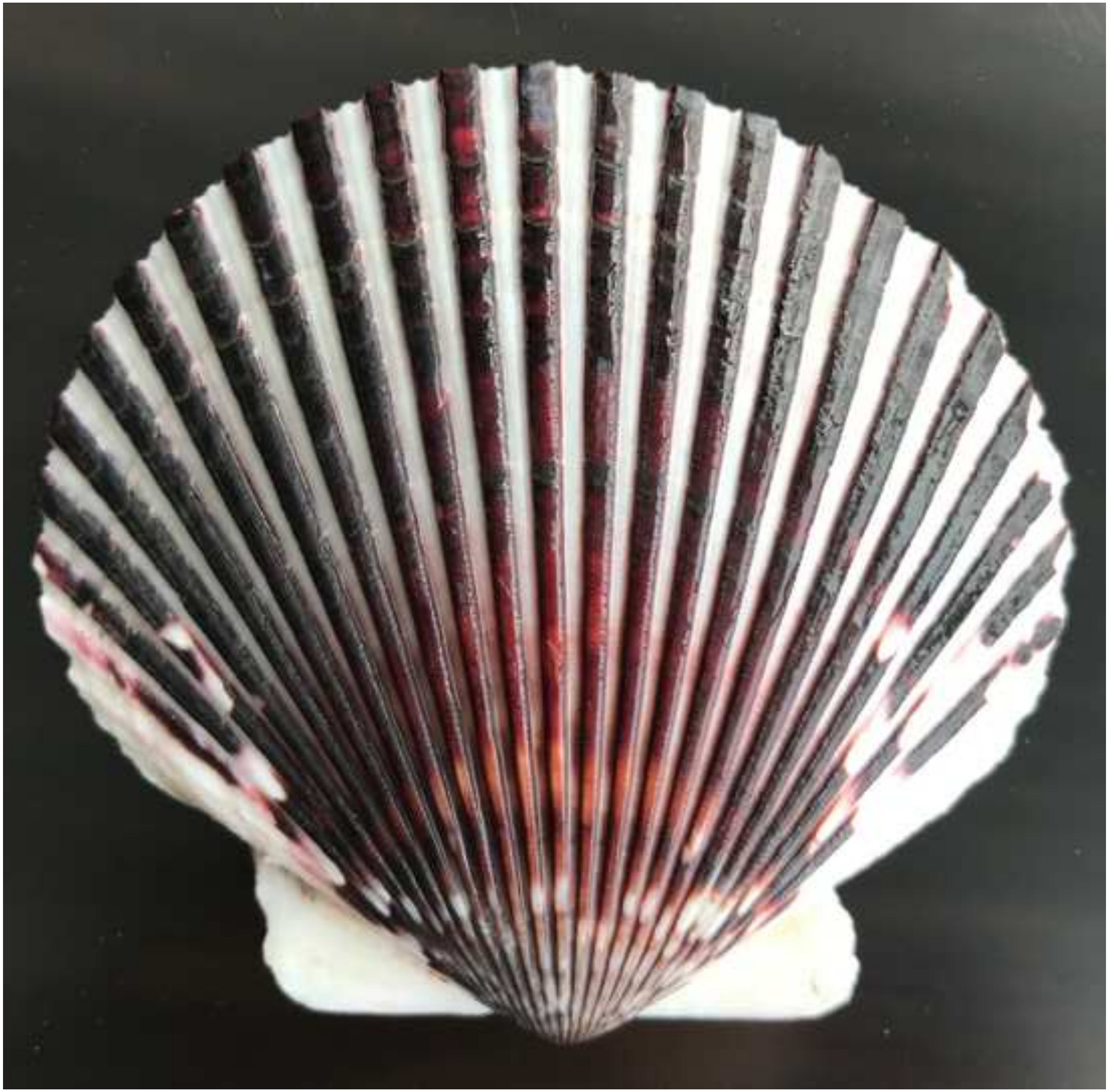

Figure

Click here to download Figure PSFig2.JPG

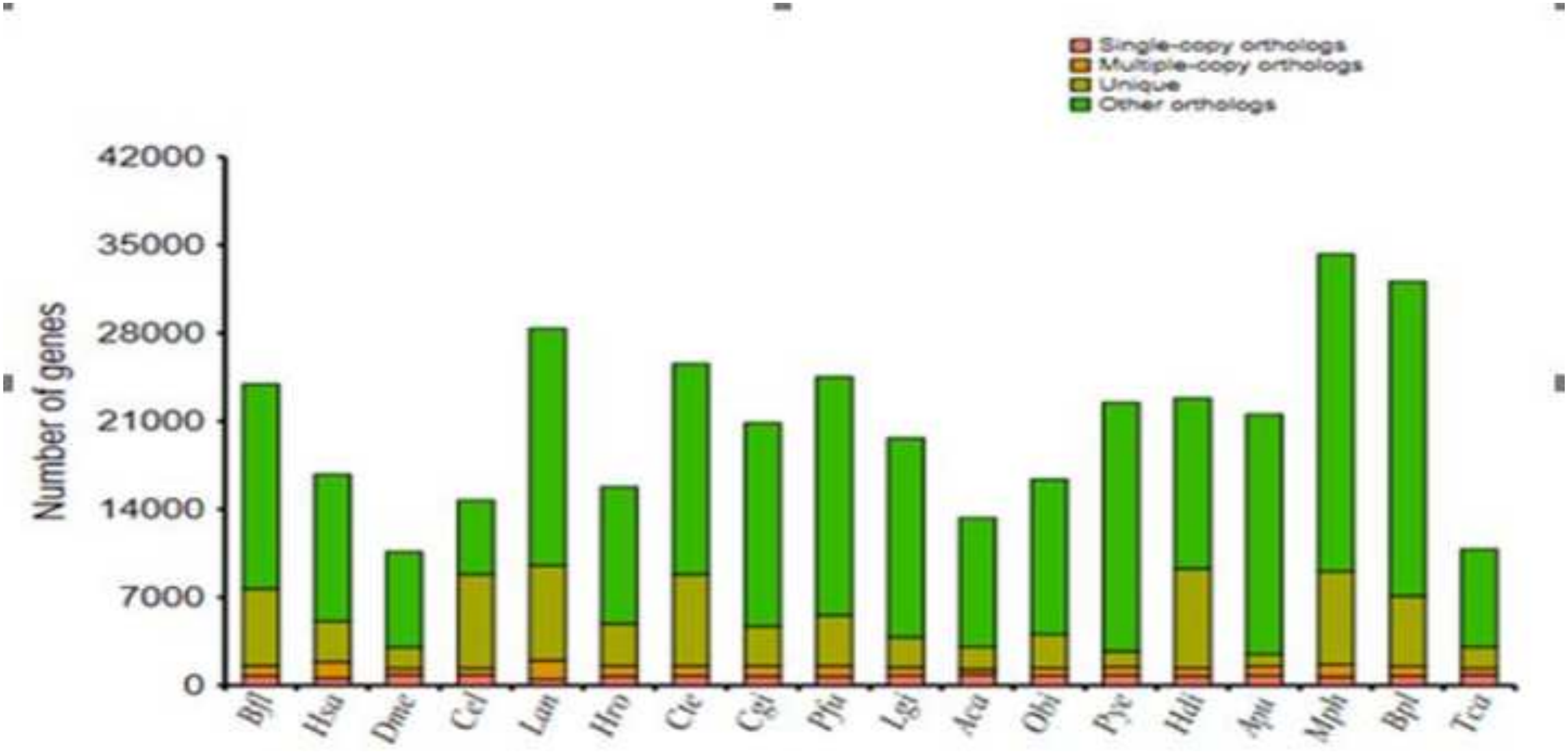

Figure

Click here to download Figure PSFig3.png

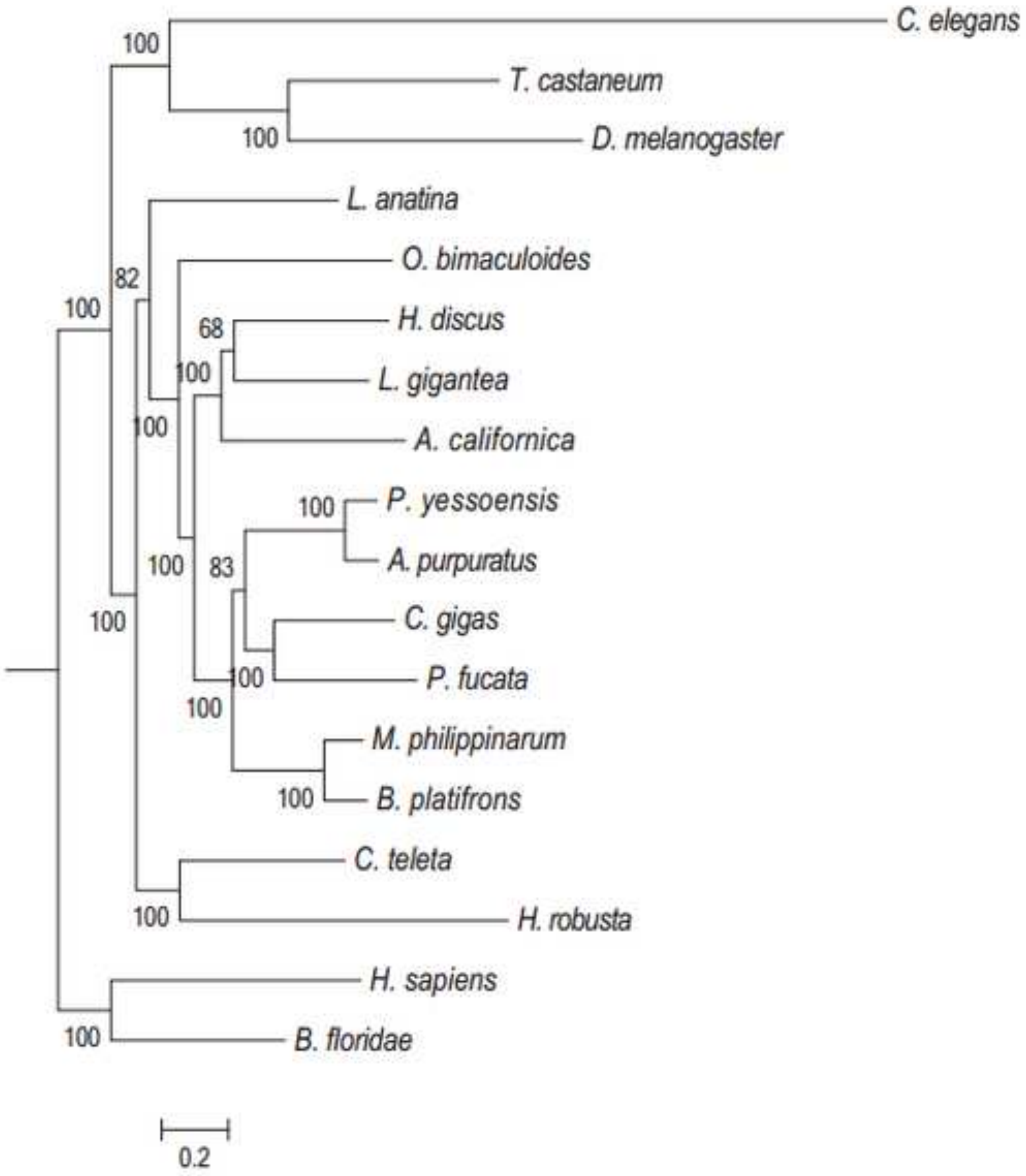

Supplement: GIGA-D-17-00315_Revision_2.pdf [file giy031_giga-d-17-00315_revision_2.pdf]
