# Supplementary material for: Draft genome of the Peruvian scallop Argopecten purpuratus
Source: Gigascience. 2018 Apr 2;7(4):giy031. doi: 10.1093/gigascience/giy031 (PMC5905365; doi:10.1093/gigascience/giy031)
Supplement: Response_to_Reviewer_Comments_Original_Submission.pdf [file giy031_response_to_reviewer_comments_original_submission.pdf]

Author's Response To Reviewer Comments

00000000-0000-(	6kmmfXrqShyu85
E2A9317A	mllKNOMSrkNiO5

Close

Dear Editor,
Thanks for your kindly consideration of publication of our manuscript in the journal. We appreciate the insightful comments from the anonymous reviewers very much. I have inlcuded the response letter in the supplementary file as the figures will not show up in this box. If you have more questions, please feel free to let us know in your earliest convenience.

Best regards,

Chao and Chunde

Close
