# Supplementary material for: Draft genome of the Peruvian scallop Argopecten purpuratus
Source: Gigascience. 2018 Apr 2;7(4):giy031. doi: 10.1093/gigascience/giy031 (PMC5905365; doi:10.1093/gigascience/giy031)
Supplement: Response_to_Reviewer_Comments_Revision_1.pdf [file giy031_response_to_reviewer_comments_revision_1.pdf]

Author's Response To Reviewer Comments

00000000-0000-(	unw4cuDnNMQx
E2A9317A	ik6twWix9bDJzm

Close

Dear Editor,
Thank you for the comments on the revised manuscript. We have corrected the manuscript thoroughly and have responded to the Reviewer's comments as attached below.
If you have more questions about the re-revised manuscript, please feel free to let us know. Thanks again for your kindly consideration of our paper.

Best regards,

Chunde and Chao

Reviewer reports:
Reviewer #2: I found all major questions by the reviewers were answered and sufficient information and data were added in the revised manuscript.
Now I would like to ask the authors to carefully correct some typos.

There are still uncorrected typos which the reviewers pointed out.
Please check the WHOLE sentences again and correct them.
Response: The manuscript has been revised carefully.

Lines 161-164 and Lines 207-212
Description of animal common names are inconsistent.
For example, "Molluscs", "Mosquito"... should be decapitalized.
Response: The names have been corrected.

Some are singular form and others are plural, which should be consistent.
i.e. "molluscs", "mosquitoes"... or "mollusca", "mosquito"...
Response: The names have been corrected!

In addition, in line 161 "Molluscs" while in line 210 "mollusk". They should be consistent.
Response: Corrected.

Line 210
"Lottia gigantean" should be "Lottia gigntea"
Response: Corrected.

Line 248
Mya stands for "million years ago." So "Mya ago" should be "Mya."
Response: Corrected.

Close
