# Supplementary material for: Draft genome of the Peruvian scallop Argopecten purpuratus
Source: Gigascience. 2018 Apr 2;7(4):giy031. doi: 10.1093/gigascience/giy031 (PMC5905365; doi:10.1093/gigascience/giy031)
Supplement: Reviewer_1_Report_(Original_Submission) -- Kevin Kocot — 12/29/2017 Reviewed [file giy031_reviewer_1_report_(original_submission).pdf]

Reviewer Report

Title: **Draft genome of the Peruvian scallop Argopecten purpuratus**

Version: **Original Submission** **Date:** 12/29/2017

Reviewer name: **Kevin Kocot**

Reviewer Comments to Author:

In this study, the authors sequenced and assembled the genome of the Peruvian scallop Argopecten purpuratus. The science seems sound but many important details are omitted. I don't see the relevance of the phylogenomic analysis placing scallops among metazoans to the objectives of the study. That same is true for the number of genes present in diverse metazoan genomes. Aside from a significant number of spelling, spacing, and grammatical corrections that need to be made, I have a number of questions regarding the methodology and and other details that are lacking:Line 48: insert "the" before "Chilean"Line 51: Correct "stenotherm" to "stenothermic"Line 59: Was adductor muscle used?Line 61: I suggest replacing "The traditional" with "A"Line 63: Substantially more detail is needed for library preparation than currently provided ("standard protocol"). How was the DNA sheared? Which libraries were paired-end and which libraries were mate pair? Line 66: I think there is a run-on sentence from lines 66-68. It is unclear what is meant by "to optimize for the most high-quality and longest reads" currently.Line 68: Insert a space after "(" and change "the 10X" to "a 10X"Line 76: How were the raw reads trimmed? Line 81: Why was a 17-mer selected?Line 133: Correct "Mollusks" to "molluscs" and "Lottia gigantean" to "Lottia gigantea"Line 140: No information is presented as to how the transcriptome data were generated. Was adductor muscle used?Line 210: Kocot et al. 2011 and Smith et al. 2011 (both published in Nature) would be appropriate citations here.Line 212: How was the molecular clock calibrated?

Level of Interest

Please indicate how interesting you found the manuscript: An article whose findings are important to those with closely related research interests

Quality of Written English

Please indicate the quality of language in the manuscript: Not suitable for publication unless extensively edited

Declaration of Competing Interests

Please complete a declaration of competing interests, considering the following questions:

- Have you in the past five years received reimbursements, fees, funding, or salary from an organisation that may in any way gain or lose financially from the publication of this manuscript, either now or in the future?
- Do you hold any stocks or shares in an organisation that may in any way gain or lose financially from the publication of this manuscript, either now or in the future?

- Do you hold or are you currently applying for any patents relating to the content of the manuscript?
- Have you received reimbursements, fees, funding, or salary from an organization that holds or has applied for patents relating to the content of the manuscript?
- Do you have any other financial competing interests?
- Do you have any non-financial competing interests in relation to this paper?

If you can answer no to all of the above, write 'I declare that I have no competing interests' below. If your reply is yes to any, please give details below.

I declare that I have no competing interests

I agree to the open peer review policy of the journal. I understand that my name will be included on my report to the authors and, if the manuscript is accepted for publication, my named report including any attachments I upload will be posted on the website along with the authors' responses. I agree for my report to be made available under an Open Access Creative Commons CC-BY license (http://creativecommons.org/licenses/by/4.0/). I understand that any comments which I do not wish to be included in my named report can be included as confidential comments to the editors, which will not be published.

I agree to the open peer review policy of the journal

To further support our reviewers, we have joined with Publons, where you can gain additional credit to further highlight your hard work (see: https://publons.com/journal/530/gigascience). On publication of this paper, your review will be automatically added to Publons, you can then choose whether or not to claim your Publons credit. I understand this statement.

Yes
