# Supplementary material for: Draft genome of the Peruvian scallop Argopecten purpuratus
Source: Gigascience. 2018 Apr 2;7(4):giy031. doi: 10.1093/gigascience/giy031 (PMC5905365; doi:10.1093/gigascience/giy031)
Supplement: Reviewer_2_Report_(Original_Submission) -- Takeshi Takeuchi, Ph.D — 1/4/2018 Reviewed [file giy031_reviewer_2_report_(original_submission).pdf]

Reviewer Report

Title: Draft genome of the Peruvian scallop Argopecten purpuratus

Version: Original Submission **Date:** 1/4/2018

Reviewer name: Takeshi Takeuchi, Ph.D

Reviewer Comments to Author:

In the manuscript Li et al. decoded the draft genome of the Peruvian scallop and demonstrated initial analyses of the genome. The data provide fundamental genomic resource to develop aquaculture of this species. I believe the data is essential to understand the biology of the species and worth to be published in the journal GigaScience after some minor revisions. I would like to ask for some more information in order to confirm the reliability and reproducibility of the data analyses. Lines 80-86Although the authors conducted k-mer analysis, the result was not shown in the manuscript. In general, bivalve genomes are high rate of heterozygosity and two peaks are appeared in k-mer analysis. Therefore, I would request the authors to show the histogram of the k-mer frequency distribution in order to see how they determined the peak k-mer depth was 69.In addition, the heterozygosity in the genome considerably affects the quality of the assembly because the assembly may contain a lot of redundant sequences derived from heterozygous loci. In order to confirm the reliability of the assembly, it is necessary to show whether the genome is heterozygous or not, and if it is true, the authors should explain how they solve the issue of heterozygosity in the sequencing and assembling processes. Lines 212-218For the divergence time estimation, the authors should describe which point was used as calibration of the time (the calibration should be based on the fossil records and their references should be listed of course). Minor pointsLine 61What does "traditional" mean here? whole genome shotgun sequence using massive parallel sequencers such as Illumina HiSeq has been applied over the past 10 years. It is not appropriate to say such a relatively recent technology as "traditional".The authors should specify which version of the software (GapCloser, fragScaff, BWA, etc.) they used. This information is necessary for replication study.Typos in scientific names"Lottia gigantean" should be "Lottia gigantea""Lingula anatine" should be "Lingula anatina"

Level of Interest

Please indicate how interesting you found the manuscript: An article whose findings are important to those with closely related research interests

Quality of Written English

Please indicate the quality of language in the manuscript: Acceptable

Declaration of Competing Interests

Please complete a declaration of competing interests, considering the following questions:

- Have you in the past five years received reimbursements, fees, funding, or salary from an organisation that may in any way gain or lose financially from the publication of this manuscript, either now or in the future?

- Do you hold any stocks or shares in an organisation that may in any way gain or lose financially from the publication of this manuscript, either now or in the future?
- Do you hold or are you currently applying for any patents relating to the content of the manuscript?
- Have you received reimbursements, fees, funding, or salary from an organization that holds or has applied for patents relating to the content of the manuscript?
- Do you have any other financial competing interests?
- Do you have any non-financial competing interests in relation to this paper?

If you can answer no to all of the above, write 'I declare that I have no competing interests' below. If your reply is yes to any, please give details below.

I declare that I have no competing interests.

I agree to the open peer review policy of the journal. I understand that my name will be included on my report to the authors and, if the manuscript is accepted for publication, my named report including any attachments I upload will be posted on the website along with the authors' responses. I agree for my report to be made available under an Open Access Creative Commons CC-BY license (http://creativecommons.org/licenses/by/4.0/). I understand that any comments which I do not wish to be included in my named report can be included as confidential comments to the editors, which will not be published.

I agree to the open peer review policy of the journal

To further support our reviewers, we have joined with Publons, where you can gain additional credit to further highlight your hard work (see: https://publons.com/journal/530/gigascience). On publication of this paper, your review will be automatically added to Publons, you can then choose whether or not to claim your Publons credit. I understand this statement.

Yes
