# Supplementary material for: Draft genome of the Peruvian scallop Argopecten purpuratus
Source: Gigascience. 2018 Apr 2;7(4):giy031. doi: 10.1093/gigascience/giy031 (PMC5905365; doi:10.1093/gigascience/giy031)
Supplement: Reviewer_2_Report_(Revision_1) -- Takeshi Takeuchi, Ph.D — 2/14/2018 Reviewed [file giy031_reviewer_2_report_(revision_1).pdf]

Reviewer Report

Title: Draft genome of the Peruvian scallop Argopecten purpuratus

Version: Revision 1 **Date:** 2/14/2018

Reviewer name: Takeshi Takeuchi, Ph.D

Reviewer Comments to Author:

I found all major questions by the reviewers were answered and sufficient information and data were added in the revised manuscript.
Now I would like to ask the authors to carefully correct some typos.

There are still uncorrected typos which the reviewers pointed out.
Please check the WHOLE sentences again and correct them.

Lines 161-164 and Lines 207-212
Description of animal common names are inconsistent.
For example, "Molluscs", "Mosquito"... should be decapitalized.
Some are singular form and others are plural, which should be consistent.
i.e. "molluscs", "mosquitoes"... or "mollusca", "mosquito"...

In addition, in line 161 "Molluscs" while in line 210 "mollusk". They should be consistent.

Line 210
"Lottia gigantean" should be "Lottia gigntea"

Line 248
Mya stands for "million years ago." So "Mya ago" should be "Mya."

Level of Interest

Please indicate how interesting you found the manuscript: An article of importance in its field

Quality of Written English

Please indicate the quality of language in the manuscript: Needs some language corrections before being published

Declaration of Competing Interests

Please complete a declaration of competing interests, considering the following questions:

- Have you in the past five years received reimbursements, fees, funding, or salary from an organisation that may in any way gain or lose financially from the publication of this manuscript, either now or in the future?
- Do you hold any stocks or shares in an organisation that may in any way gain or lose financially from the publication of this manuscript, either now or in the future?

- Do you hold or are you currently applying for any patents relating to the content of the manuscript?
- Have you received reimbursements, fees, funding, or salary from an organization that holds or has applied for patents relating to the content of the manuscript?
- Do you have any other financial competing interests?
- Do you have any non-financial competing interests in relation to this paper?

If you can answer no to all of the above, write 'I declare that I have no competing interests' below. If your reply is yes to any, please give details below.

I declare that I have no competing interests

I agree to the open peer review policy of the journal. I understand that my name will be included on my report to the authors and, if the manuscript is accepted for publication, my named report including any attachments I upload will be posted on the website along with the authors' responses. I agree for my report to be made available under an Open Access Creative Commons CC-BY license (http://creativecommons.org/licenses/by/4.0/). I understand that any comments which I do not wish to be included in my named report can be included as confidential comments to the editors, which will not be published.

I agree to the open peer review policy of the journal

To further support our reviewers, we have joined with Publons, where you can gain additional credit to further highlight your hard work (see: https://publons.com/journal/530/gigascience). On publication of this paper, your review will be automatically added to Publons, you can then choose whether or not to claim your Publons credit. I understand this statement.

Yes
